# The role of the SWI/SNF chromatin remodeling complex in maintaining the stemness of glioma initiating cells

**DOI:** 10.1038/s41598-017-00982-3

**Published:** 2017-04-18

**Authors:** Hiroaki Hiramatsu, Kazuyoshi Kobayashi, Kyousuke Kobayashi, Takeshi Haraguchi, Yasushi Ino, Tomoki Todo, Hideo Iba

**Affiliations:** 1 Division of Host-Parasite Interaction, Department of Microbiology and Immunology, The Institute of Medical Science, The University of Tokyo, Tokyo, 108-8639 Japan; 2 Division of RNA Therapy, Medical Mycology Research Center, Chiba University, Chiba, 260-8673 Japan; 3 Division of Innovative Cancer Therapy, and Department of Surgical Neuro-Oncology, The Institute of Medical Science, The University of Tokyo, Tokyo, 108-8639 Japan

## Abstract

Glioma initiating cells (GICs) are thought to contribute to therapeutic resistance and tumor recurrence in glioblastoma, a lethal primary brain tumor in adults. Although the stem-like properties of GICs, such as self-renewal and tumorigenicity, are epigenetically regulated, the role of a major chromatin remodeling complex in human, the SWI/SNF complex, remains unknown in these cells. We here demonstrate that the SWI/SNF core complex, that is associated with a unique corepressor complex through the d4-family proteins, DPF1 or DPF3a, plays essential roles in stemness maintenance in GICs. The serum-induced differentiation of GICs downregulated the endogenous expression of *DPF1* and *DPF3a*, and the shRNA-mediated knockdown of each gene reduced both sphere-forming ability and tumor-forming activity in a mouse xenograft model. Rescue experiments revealed that DPF1 has dominant effects over DPF3a. Notably, whereas we have previously reported that d4-family members can function as adaptor proteins between the SWI/SNF complex and NF-κB dimers, this does not significantly contribute to maintaining the stemness properties of GICs. Instead, these proteins were found to link a corepressor complex containing the nuclear receptor, TLX, and LSD1/RCOR2 with the SWI/SNF core complex. Collectively, our results indicate that DPF1 and DPF3a are potential therapeutic targets for glioblastoma.

## Introduction

Glioblastoma multiforme (GBM) is the most common malignant brain tumor in adults and remains incurable in spite of aggressive treatment approaches. A large body of evidence now indicates that stem-like cells, designated as glioma initiating cells (GICs), are thought to drive GBM propagation and cause therapeutic resistance in these tumors^1–3^. Chromatin structure modification has been shown to be an important determinant of GIC stemness maintenance as well as the induction of their differentiation^4, 5^. Recently, using gene expression data from both stem-like and differentiated cell populations, it was shown that the simultaneous expression of four core transcription factors, POU3F2, SALL2, SOX2 and OLIG2, can reprogram differentiated GBM cells into spherogenic stem-like tumor-propagating cells^6^. These results demonstrate a plastic developmental hierarchy in GBM cell populations and reveal essential roles of epigenetic regulation in these biological processes^7^.

In humans, the ATP-dependent chromatin remodeling factor, SWI/SNF complex, has been reported to play essential epigenetic roles in many biological processes^8–10^. As the catalytic subunit, each SWI/SNF complex has a single molecule of either BRG1 or Brm, but not both. The molecular components of these SWI/SNF complexes are now known to be highly polymorphic, in which some subunits that are encoded by homologous gene family members are integrated into the specific position of the complex in a mutually exclusive manner^8, 9, 11^. Importantly, exchange of a subunit with another family member has often been observed during several developmental processes within either the SWI/SNF core complex or its strongly associated cofactor proteins. Some of those exchanges are anticipated to be crucial for these developmental transitions. For example, BRG1 is much more abundant than Brm in embryonic and neural stem cells and is thus thought to be the major functional catalytic subunit in these cellular contexts^8, 9, 12–14^. In a similar mutually exclusive manner, one of DPF1 (BAF45B), DPF2 (REQ/BAF45D), DPF3 (BAF45C), which comprise the d4-family proteins, is a cofactor that substoichiometrically interacts with human SWI/SNF complexes.

We have previously demonstrated that DPF2 functions as an efficient adaptor protein between the SWI/SNF complex and the RelB/p52 dimer^15^. By examining all of the d4-family proteins (DPF1, DPF2, DPF3a and DPF3b; a splicing variant of DPF3a), we further found that high level exogenous expression of each of these factors can potentiate the transactivating activity of typical NF-κB dimers including RelA/p50, which is responsible for the canonical NF-κB pathway, and RelB/p52, which is the most downstream factor of the non-canonical NF-κB pathway^16^. In addition, we demonstrated from our analysis in 293FT cells that DPF3a and 3b are the most effective cofactors of the SWI/SNF complex for RelA/p50 activation.

In our current study, we show that knockdown of either *DPF1* or *DPF3a* promptly abolishes stemness maintenance of GICs. We have demonstrated that through these d4-family proteins, a distinct SWI/SNF core complex is associated with specific corepressor complexes and further that such larger SWI/SNF complex is an essential determinant of the key features of GICs. Therefore, DPF1 and DPF3a would be possible new therapeutic targets for GBM.

## Results

### *DPF1* and *DPF3a/b* transcripts are abundant in GICs in sphere cultures but are downregulated upon differentiation

**Figure 1:**
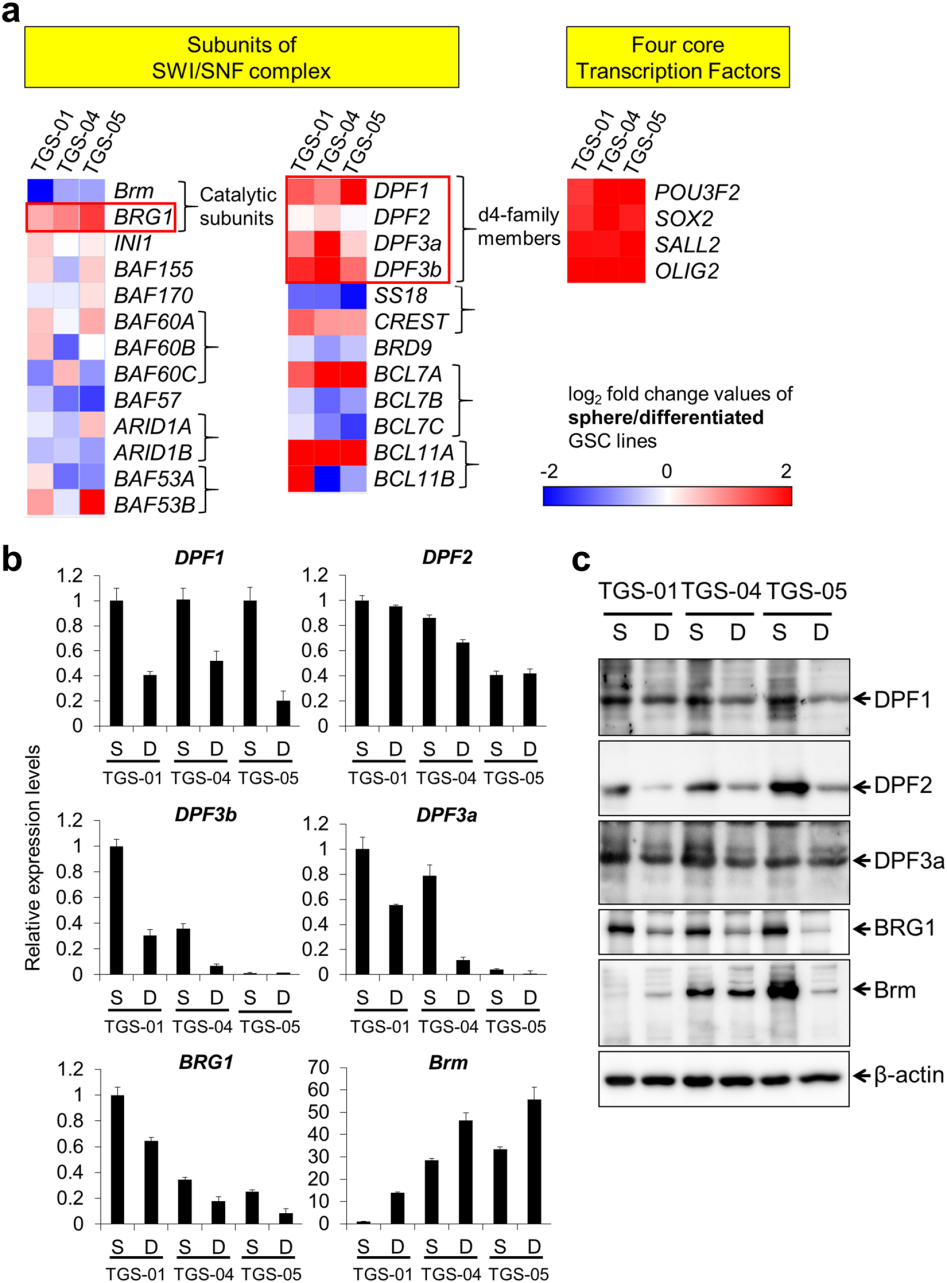
Expression of mRNAs and proteins of the subunits of SWI/SNF core complex in three GIC preparations and their corresponding differentiated cells. mRNA (**a**,**b**) and protein (**c**) expression in sphere cultures of TGS-01, -04 or -05 were analyzed by qRT-PCR and western blotting, respectively, and compared with those in differentiated monolayer cultures derived from these cultures. (**a**) The heat map represents the log2 fold changes in gene expression (sphere culture/differentiated monolayer culture). Red and blue indicate higher and lower expression, respectively, in sphere cultures compared with differentiated monolayer cultures. (**b**) Relative gene expression levels of the d4-family members, *BRG1* and *Brm* were analyzed by qRT-PCR using the same RNA described in (**a**). Error bars represent standard deviation of the mean from triplicate experiments. (**c**) Protein expression levels of the d4-family members, BRG1 and Brm were analyzed by western blotting. The same set of protein samples was used for each blot in which equal amounts and β-actin was used as the loading control. S, sphere culture; D, differentiated monolayer culture.

Three independent cell isolates from GBM patients (TGS-01, -04 and -05) have been reported previously to have stem cell-like properties when cultured as non-adherent spheres in a defined medium without serum^17^. The differentiation of these three GIC isolates can be induced by culturing the cells as an adherent monolayer in medium containing serum^18^. We isolated total RNA and protein from 3 respective pairs of sphere and differentiated monolayer GIC cultures. By qRT-PCR and western blotting using our GIC preparations, we checked the expression of the POU3F2, SOX2, SALL2 and OLIG2, which have been reported to be required for the reconstitution and maintenance of stemness^6^ (Fig. 1a, and Supplementary Fig. S1). By comparing the RNA and protein levels between the sphere and differentiated monolayer GIC cultures, we found that all 4 transcription factors were at higher levels in sphere culture, indicating that these GIC cultures had very similar properties to stem-like tumor propagating cells (TPCs) reported previously^6^.

Using the same samples, we next examined the expression levels of core components of SWI/SNF complex and of several proteins reported to be strongly associated with SWI/SNF complex. The mRNA levels of *BRG1*, the catalytic subunit thought to be involved in the stemness maintenance in embryonic and neuronal stem cells, were higher in sphere cultures than in differentiated cultures of all three GICs (Fig. 1a,b). Interestingly, among the d4-family members, *DPF1* and *DPF3a/b* were found to be more abundant in sphere cultures (Fig. 1a), although the expression levels of *DPF3a/b* varied among the three GIC sphere cultures (Fig. 1b). We next analyzed protein expression levels of d4-family proteins, BRG1 and Brm by western blotting (Fig. 1c). Reflecting the mRNA levels, protein levels of DPF1, DPF3a and BRG1 were higher in sphere cultures of these cells. Unlike mRNA levels, however, the levels of DPF2 protein were much higher in sphere cultures than in differentiated cultures.

Whereas the higher expression levels of *Brm* mRNA in differentiated cultures were basically similar among the three GICs cultures, we observed that the *Brm* mRNA levels in sphere cultures of TGS-04 and TGS-05 were considerably higher than in the TGS-01 cultures (Fig. 1b). When Brm proteins in TGS-04 and TGS-05 cells were analyzed by western blotting (Fig. 1c), unlike mRNA levels, the protein levels were higher in sphere cultures than in differentiated cultures, indicating that there would be some post-transcriptional regulation of Brm expression in these cells. These results also suggest a significant heterogeneity in Brm expression among the three GIC cultures.

### DPF1 and DPF3a play important roles in maintaining GIC stemness

**Figure 2:**
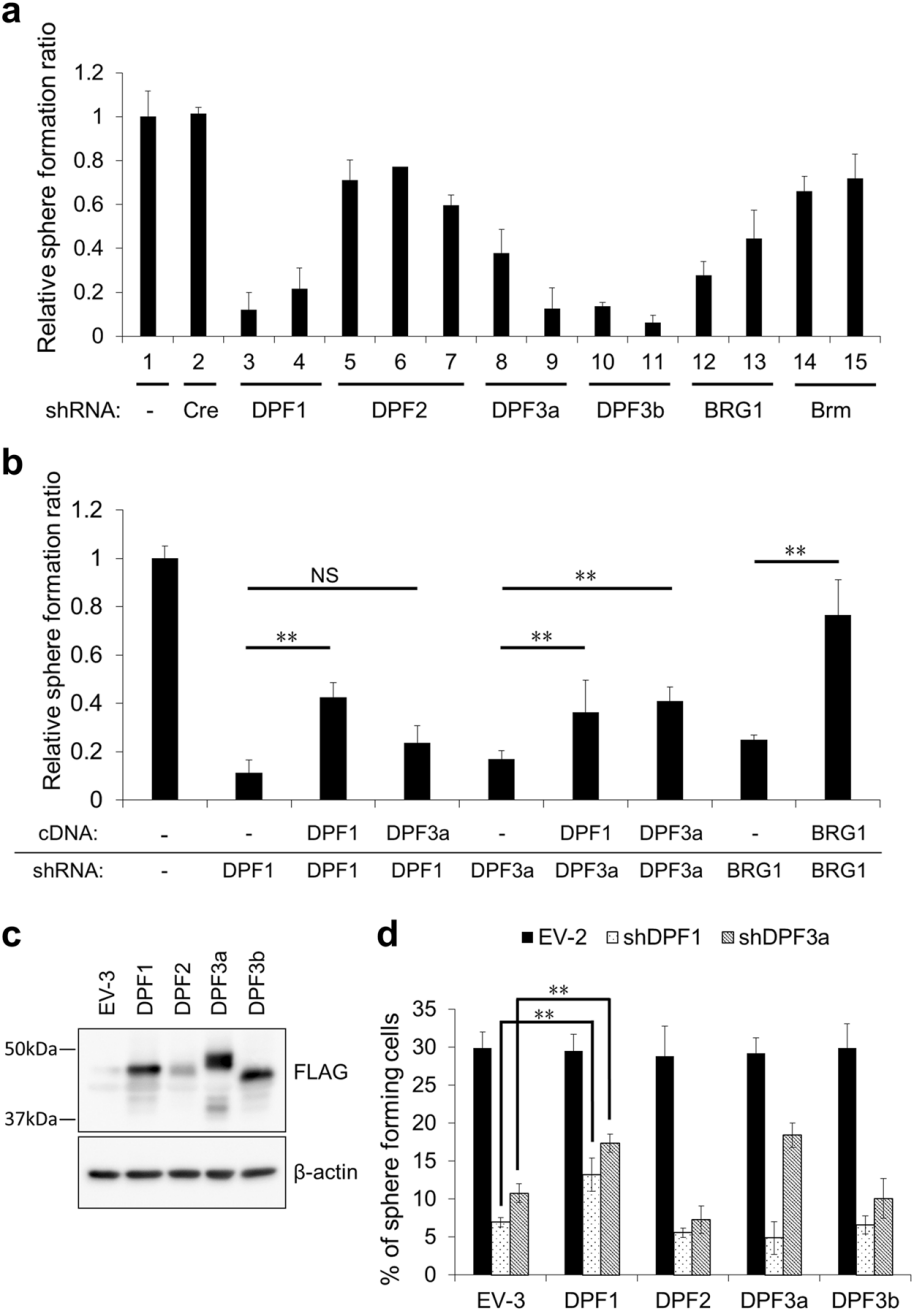
Knockdown effects of d4-family members, *BRG1* or *Brm* on the sphere forming activity of TGS-01 cells. (**a**) Relative sphere formation ratio of TGS-01 cells transduced with retrovirus vectors expressing various shRNAs or empty vector (EV-1; lane 1); shCre#4 (lane 2), shDPF1-CDS#1 (lane 3), shDPF1-3′UTR#4 (lane 4), shDPF2-3′UTR#3, #4 and #6 (lanes 5–7), shDPF3a-3′UTR#2 (lane 8), and shDPF3a-3′UTR#4 (lane 9), shDPF3b-CDS#6 and #7 (lanes 10, 11), shBRG1-CDS#2 and #4 (lanes 12, 13) and shBrm#4, and #8 (lanes 14, 15). (**b**) Relative sphere formation ratio of TGS-01 cells transduced with the dual lentivirus vectors based on pLE-IG simultaneously expressing mRNA (3 × FLAG-DPF1, -DPF3a and -BRG1) and shRNA (shCre#4, shDPF1-3′UTR#4, shDPF3a-3′UTR#4, shBRG1-CDS#2) for the rescue experiments. (**c**) TGS-01 cells transduced with lentivirus vectors based on pLE-IP expressing d4-family proteins as well as an empty vector (EV-3) were prepared. Total proteins were prepared from them and analyzed by western blotting using anti-FLAG antibody. β-actin was used as the loading control. The position of protein markers (#161-0374; BIO-RAD) were indicated. (**d**) Parallel cultures prepared in (**c**) were subsequently transduced with lentivirus vectors based on pLE-IG expressing shDPF1-3′UTR#4, shDPF3a-3′UTR#4, or shBRG1-CDS#2 as well as an empty vector (EV-2). Error bars represent standard deviation of the mean from triplicate experiments. NS = not significant, *p < 0.05, **p < 0.01 by Student’s t-test.

To test the possible involvement of the d4-family proteins and BRG1 in stem cell maintenance in GICs, we performed respective knockdown experiments using at least two sets of short-hairpin (sh)RNAs with efficient suppressing activity. Efficiency and specificity of these shRNAs were confirmed in our previous work^19^ or in this current study (Supplementary Fig. S2) according to the following criteria: mRNA levels of each target were specifically reduced to at least 40% in 293FT or MDA-MB-231 cells without affecting the other member of the family gene. Our preliminary experiments indicated that the biological effects of a *DPF1* and *DPF3a* knockdown seemed to be so rapid that we could not isolate stable transductants in non-adherent sphere culture by puromycin selection. We therefore employed shRNA expression lentivirus vectors coexpressing GFP. To evaluate the stemness of GICs, we performed sphere forming assays in which GFP positive cells were single-sorted into a well at 48 hours after transduction and sphere forming activity was evaluated. The knockdown of *BRG1*, *DPF1*, *DPF3a*, and *DPF3b* drastically reduced the sphere forming activity of TGS-01, whereas a *DPF2* knockdown showed only marginal effects in this assay (Fig. 2a). To test the function of d4-family proteins in the other GICs, we subjected TGS-04 and TGS-05 cells to *DPF1*, *DPF2, DPF3a* or *DPF3b* knockdown and found that sphere forming activity was reduced specifically by the knockdown of *DPF1* and *DPF3a*, (Supplementary Fig. S3), underscoring the general importance of DPF1 and DPF3a in the maintenance of GICs stemness.

To eliminate the possibility of off-target effects by the shRNA constructs, rescue experiments were performed. Since the loss of stemness can be detected very early after vector transduction, we designed and developed dual lentivirus expression vectors (Supplementary Fig. S4) carrying expression units of both shRNAs (driven by the *mU6* pol III promoter) and the corresponding cDNA (driven by the *EF1α* pol II promoter known to be relatively resistant to gene silencing in stem cells). The cDNAs were designed to be resistant to the corresponding shRNA; when shRNA detects 3′-UTR, cDNA was designed not to including the 3′-UTR, and when shRNA detects the coding sequence, the target site was mutated without changing amino acid sequence. In the case of *BRG1*, *DPF1* and *DPF3a*, the simultaneous expression of the corresponding cDNA insensitive to the corresponding shRNA partially rescued the sphere forming activity (Fig. 2b). Interestingly, *DPF3a* knockdown was also rescued by DPF1 cDNA expression, whereas *DPF1* knockdown was not significantly rescued by the exogenous expression of DPF3a (Fig. 2b). These results suggest that DPF1 has dominant effects over DPF3a in terms of stem cell maintenance. Because rescue experiments using DPF3b cDNA in *DPF3b* knockdown cells were not successful and also because the mRNA levels of *DPF3b* are close to the limit of detection by qRT-PCR in these GICs, we did not further analyze *DPF3b*. It was notable that the exogenous expression of FLAG-tagged d4-family proteins, BRG1 and Brm in TGS-01 cells did not increased sphere forming activity (Supplementary Fig. S5a), whereas all of the exogenous proteins were shown to be successfully expressed (Supplementary Fig. S5b). These results indicate that the endogenous levels of these proteins were at saturated levels sufficient to maintain stemness. The extent of rescues using dual expression vectors were only partial. These results might reflect the prompt effects of shRNAs which appeared before the exogenous protein expression reached stable levels. To exclude this possibility, we prepared TGS-01 cells stably expressing the each d4-family proteins in advance (Fig. 2c) and then additionally transduced with shRNA expression vectors. As shown in Fig. 2d, when shDPF1 and shDPF3a were introduced into cells expressing the corresponding cDNA, recovery of the sphere forming activity was again partial. Moreover, when shDPF3a was expressed by additional virus vector, DPF1-introduced cells showed higher sphere forming activity compared with EV-2-introduced cells did. On the other hand, cells exogenously expressing DPF3a were found to be sensitive to the additional *DPF1* knockdown (Fig. 2d). These results confirmed our previous observations with dual expression vectors, i.e. that DPF1 is dominant over DPF3a.

As for the reason for the incomplete rescue, we believe it would be mainly explained by the fact that the exogenously introduced expression vectors for cDNA are tend to be silenced in stem-like cells. Whereas *EF1α* promoter, which we used to drive cDNA from lentivirus vector in this study, has been reported to be relatively resistant to such gene silencing^20^, it was shown to be gradually silenced in neuronal stem cells^21^.

### BRG1 can be functionally substituted by Brm as a catalytic subunit of the SWI/SNF core complex that contributes to GIC stemness

Whereas the higher expression levels of *Brm* mRNA in differentiated cultures were basically similar among the three GICs cultures, we observed that the *Brm* mRNA levels in sphere cultures of TGS-04 and TGS-05 were considerably higher than in the TGS-01 cultures (Fig. 1b). When Brm proteins in TGS-04 and TGS-05 cells were analyzed by western blotting (Fig. 1c), unlike mRNA levels, the protein levels were higher in sphere cultures than in differentiated cultures, indicating that there would be some post-transcriptional regulation of Brm expression in these cells. These results also suggest a significant heterogeneity in the SWI/SNF components among the three GIC cultures.

**Figure 3:**
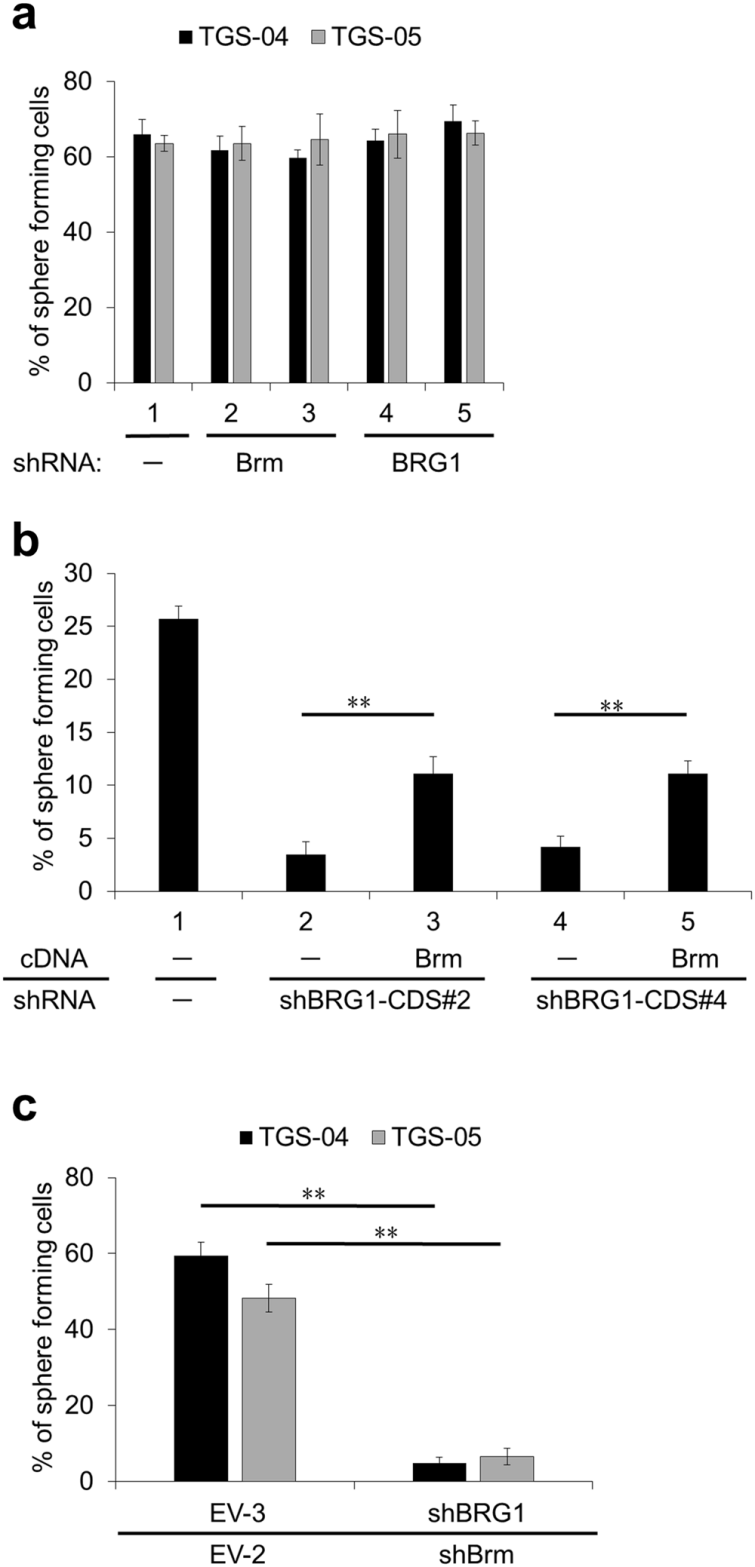
Importance of BRG1 and Brm on the sphere forming activity of GICs. (**a**) Percentage of sphere forming cells in TGS-04 and TGS-05 cultures transduced with lentivirus vectors based on pLE-IG expressing shBrm or shBRG1 or empty vector (EV-2; lane 1); shBrm#4, and #8 (lanes 2, 3) and shBRG1-CDS#2 and #4 (lanes 4, 5). No statistically significant differences were observed compared with the control (EV-2) (p > 0.05). (**b**) Percentage of sphere forming cells in TGS-01 cultures transduced with the dual lentivirus vectors based on pLE-IG expressing 3 × FLAG-Brm and shBRG1 simultaneously for the rescue experiments. Lanes 2 and 3 are shBRG1-CDS#2, and lanes 4 and 5 are shBRG1-CDS#4. (**c**) TGS-04 and TGS-05 cells transduced with lentivirus vectors based on pLE-IP expressing shBRG1-CDS#2 or empty vector (EV-3) were selected by puromycin, and subsequently transduced with another lentivirus vectors based on pLE-IG expressing shBrm#4 or empty vector (EV-2), respectively. Percentage of sphere forming cells in these cells were evaluated. Error bars represent standard deviation of the mean from triplicate experiments. **p < 0.01 by Student’s t-test.

We further found that the sphere forming activity of both TGS-04 and TGS-05 was insensitive to both *BRG1* and *Brm* knockdown (Fig. 3a). This finding contrasted with our observations in TGS-01 cells (Fig. 2a), in which the *BRG1* knockdown caused a reduction in sphere forming activity. We hypothesized that because of the high expression of both BRG1 and Brm proteins in TGS-04 and 05 cells, a single knockdown of either may not have significantly affected sphere forming activity. This observation raises the possibility that Brm can also function as an effective catalytic subunit of SWI/SNF complex for the stem cell maintenance of GICs.

To test whether Brm could indeed functionally substitute for BRG1, we next coexpressed Brm and shBRG1 in TGS-01 cells. The sphere forming activity in these transfectants was rescued by Brm expression (Fig. 3b), whereas the exogenous expression of Brm alone was not substantially affected the sphere forming activity (Supplementary Fig. S5a).

Importantly, TGS-04 and TGS-05 cells, the BRG1 and Brm of which were consecutively knocked down, drastically reduced the sphere forming activity (Fig. 3c). These results indicate that Brm, if expressed in GICs at high protein levels, can also contribute to the maintenance of stemness. Therefore, at least in terms of maintaining GIC stemness, the frequently observed enrichment of BRG1 in sphere cultures does not mean that Brm cannot also perform the same biological activity.

### *DPF1* and *DPF3a* knockdowns in GICs produce strong anti-tumorigenic activity

**Figure 4:**
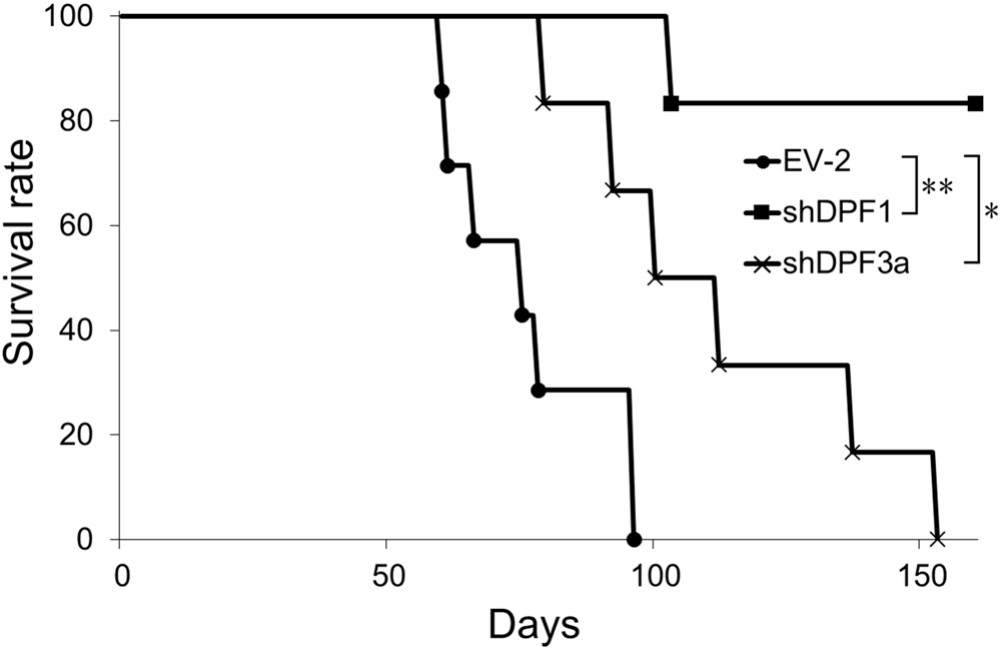
Kaplan-Meier survival curves of mice orthotopically injected with TGS-01 cells. TGS-01 cells transduced (3 × 10^3^ cells in each case) with lentivirus vectors based on pLE-IG expressing shDPF1-3′UTR#4 or shDPF3a-3′UTR#4 or empty vector (EV-2) were sorted by flow cytometry two days after the transduction and injected into the cerebral hemisphere of 6-week-old female nude mice. *p < 0.05, **p < 0.01 by log-rank test.

To further confirm the stemness maintenance function of DPF1 and DPF3a, TGS-01 cells transduced with shDPF1 or shDPF3a expression vectors as well as an EV-2 (control) cells were orthotopically inoculated into immunocompromised mice (nude mice). Although we saw a rapid reduction in the survival rate of mice inoculated with the control cells, both shDPF1 and shDPF3a expression improved this survival rate (Fig. 4). Notably, the expression of shDPF1 produced much greater effects compared with shDPF3a (5/6 mice showed full survival). These results confirmed that DPF1 has more profound effects on the maintenance of GIC stemness and thus further revealed that it has potential as a therapeutic target in GBM.

### Stemness maintenance by DPF1 and DPF3a does not require NF-κB activation

In our previous studies using epithelial tumor cell lines, the d4-family members were shown to function as adaptor proteins linking the SWI/SNF complex with NF-κB dimers^15, 16^. Considering the rather strong dependency of GIC stemness maintenance on BRG1, DPF1 and DPF3a expression, we next tested whether SWI/SNF-dependent-NF-κB activation contributed to this biological activity. When IκBαSR^22^, a mutant of the NF-κB activating pathway inhibitor IκBα, was introduced into TGS-01 cells, we did not detect any significant effects on sphere forming activity as shown in Supplementary Fig. S6a. By immunofluorescent staining of TGS-01 in sphere cultures with anti-RelA or anti-RelB antibody, we found these proteins were present mainly in the cytoplasm, indicating that NF-κB pathway is not significantly activated in GICs kept in sphere cultures (Supplementary Fig. S6). To detect possible function in nuclear NF-κB dimers, we tried PLA assay using anti-RelA/anti-BRG1 or anti-RelB/anti-BRG1 but only very low background levels of signals were detected. These results indicated that DPF1 and DPF3a must have clearly distinct functions other than adaptors between NF-κB and the SWI/SNF complex in GICs to play essential roles in the maintenance of stemness.

### Formation of a large complex comprising the SWI/SNF core complex and a corepressor complex requires DPF1 and DPF3a adaptors

**Figure 5:**
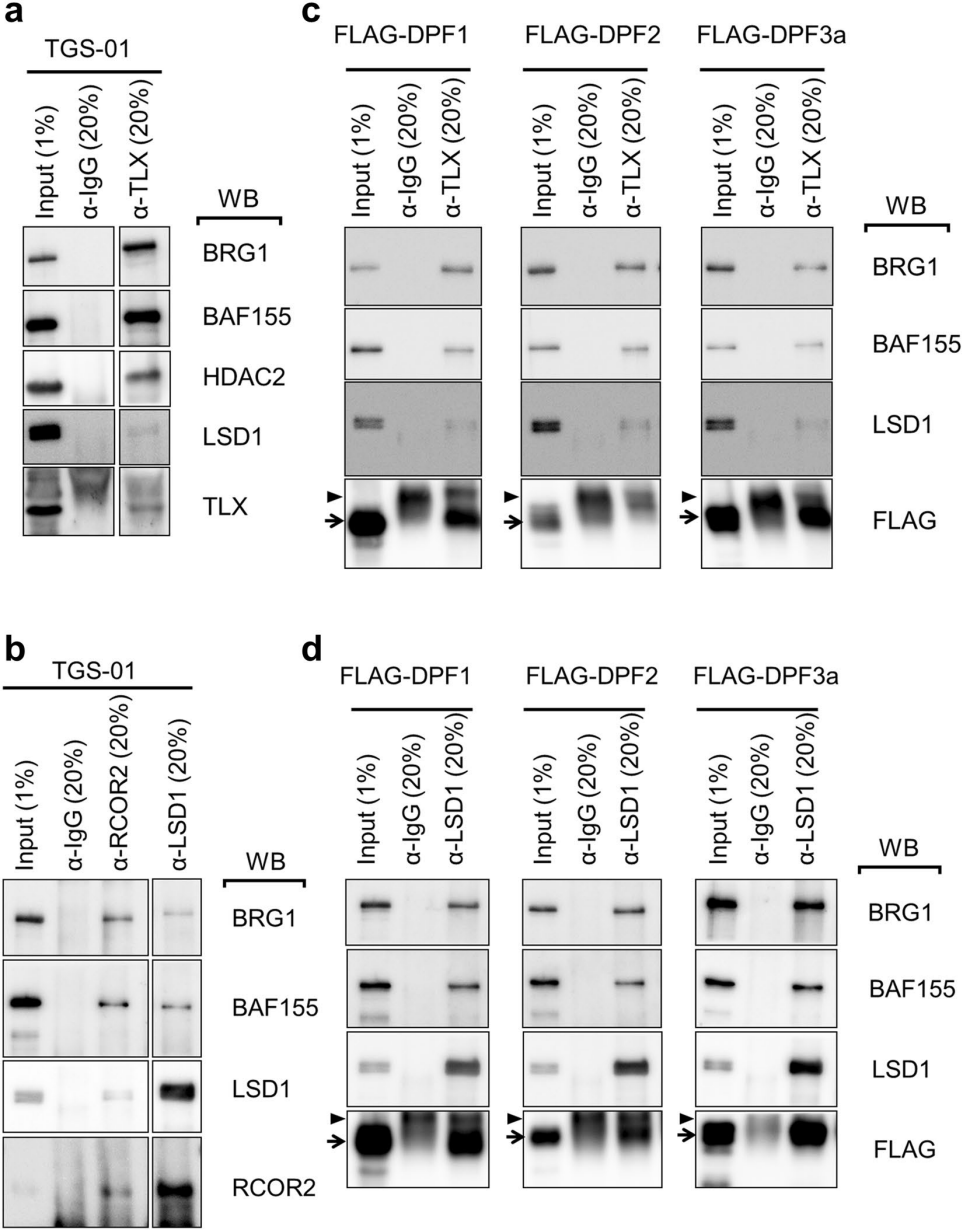
Detection of larger SWI/SNF complexes that include TLX and LSD1/RCOR2 in GICs. Coimmunoprecipitation of SWI/SNF complex subunits with corepressor complex. (**a**,**b**) TGS-01 lysates were immunoprecipitated with anti-TLX (**a**) or anti-LSD1 and anti-RCOR2 (**b**) antibodies, and the resulting immunoprecipitates were analyzed by western blotting. (**c**,**d**) TGS-01 cells exogenously expressing 3 × FLAG-DPF1, -DPF2 or -DPF3a lysates were immunoprecipitated with anti-TLX antibody (**c**) or anti-LSD1 antibody (**d**), and the immunoprecipitates were analyzed by western blotting. Arrows, FLAG-tagged d4-family proteins; arrowheads, IgG heavy chains. The samples were derive from the same experiment and the gels/blots were processed in parallel. The full-length blots were presented in the Supplementary Fig. S7.

Given our observation of the prompt and strong suppression of stemness maintenance by shDPF1, and to a lesser extent by shDPF3a, we hypothesized that DPF1 as well as DPF3a can function as adaptors between SWI/SNF core complex and key transcriptional regulators which are essential for stemness of GICs. To examine this possibility, antibodies against several candidate proteins were tested whether they are able to coimmunoprecipitate with subunits of the SWI/SNF complex. Among the possible candidates that would form large complexes with SWI/SNF core complex, we found that antibodies against TLX (NR2E1), LSD1 (lysine-specific demethylase 1) and RCOR2 (REST corepressor 2; CoREST2) coimmunoprecipitate both BRG1 and BAF155 from TGS-01 cell lysates (Fig. 5a,b; full-length images are in Supplementary Fig. S7). TLX, a nuclear orphan receptor with transcriptional suppressing function, has been previously shown to control the neuronal stem cells^23–25^ and to be essential for maintaining the stemness of GICs by knockdown experiments^26^. LSD1, a negative epigenetic regulator with histone demethylase activity, has also been reported to regulate GIC stemness in combination with RCOR2 (REST corepressor2; CoREST2), a well-known corepressor^6^. Importantly, the protein levels of TLX was unchanged after induction of differentiation of these three GIC cultures whereas those of LSD1 and RCOR2 were enriched in sphere cultures (Supplementary Fig. S1b). When TGS-01 lysates were immunoprecipitated using a TLX antibody, we detected LSD1 and HDAC2 in these immunoprecipitates (Fig. 5a). Moreover, when these lysates were immunoprecipitated with an LSD1 antibody, we detected RCOR2, and *vice versa* (Fig. 5b), confirming previous reports of tight dimer formation between these two proteins^6, 27^. Similarly, when TGS-04 and TGS-05 cellular lysates were immunoprecipitated with TLX antibody, both BRG1 and BAF155 were detected in the immunoprecipitates (Supplementary Fig. S8). Furthermore when lysates of TGS-01 cells exogenously expressing FLAG-tagged DPF1 or DPF3a were immunoprecipitated with either a TLX or LSD1 antibody, DPF1 or DPF3a as well as BRG1, BAF155 and LSD1 were detected (Fig. 5c,d; full-length images are in Supplementary Fig. S7). FLAG-tagged DPF2 was also detected but at considerably lower levels. Overall, these results suggest that DPF1 and DPF3a can function as adaptor proteins linking the SWI/SNF complex and corepressor complexes containing TLX and LSD1/RCOR2.

**Figure 6:**
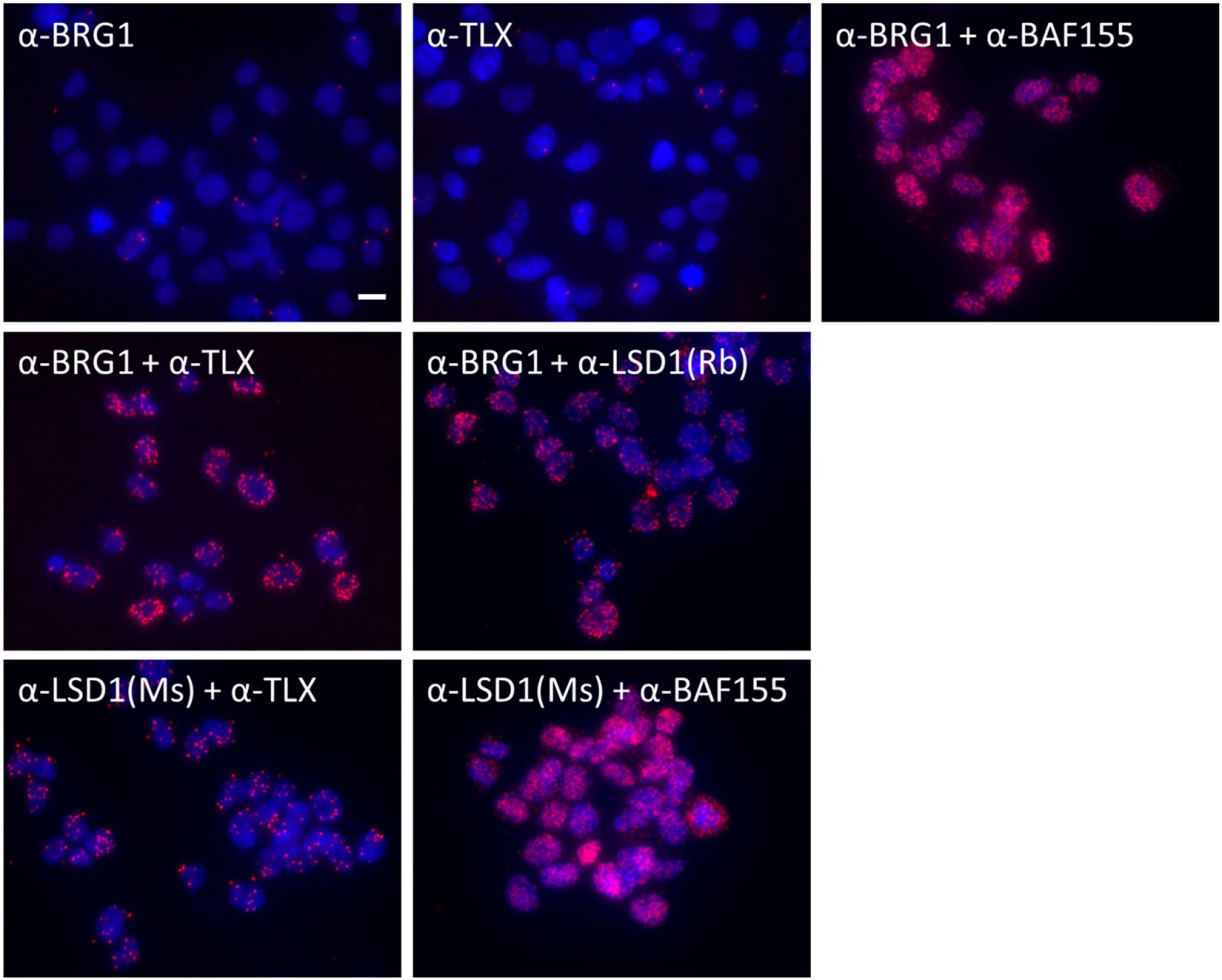
Proximal localization of the SWI/SNF core complex, TLX and LSD1/RCOR2 in TGS-01 cells as detected by PLA. TGS-01 cells were fixed and incubated with a single antibody or pairs of antibodies. Red dots indicate interactions and nuclei were counterstained with DAPI (blue). The red fluorescence images were obtained using quick-full-focus function of the BZ-X710 (Keyence) at depth of about 10 μm. Scale bar indicates 10 μm. Rb; rabbit, Ms; mouse.

**Figure 7:**
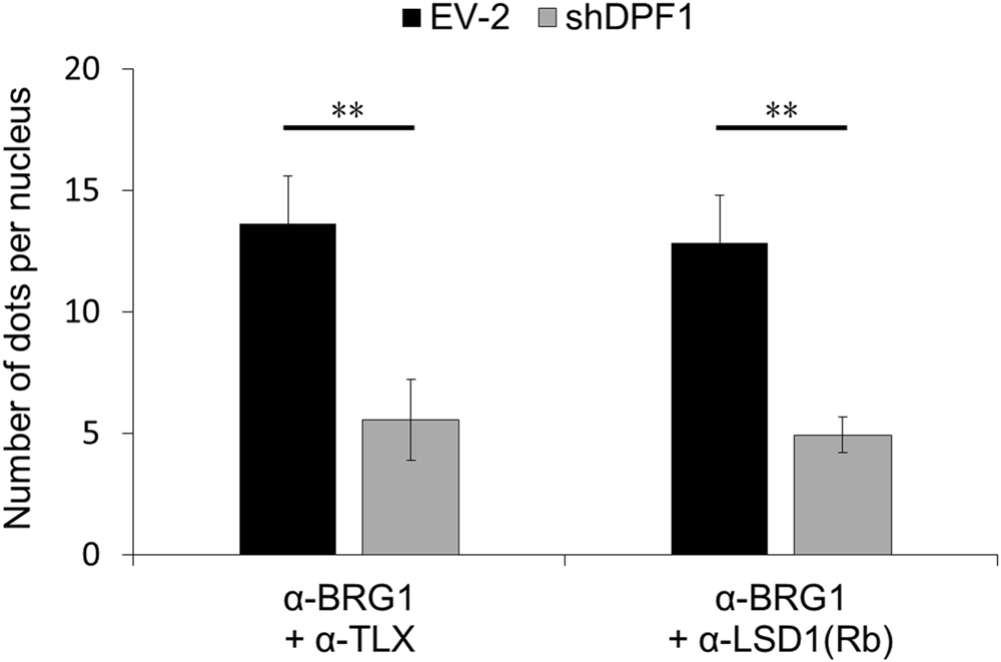
Effects of a *DPF1* knockdown on the proximal localization of BRG1 and TLX/LSD1 in TGS-01 cells as detected by PLA. TGS-01 cells transduced with lentivirus vectors based on pLE-IG expressing shDPF1-3′UTR#4 or an empty vector (EV-2) were fixed three days after the transduction and incubated with pairs of antibody. The number of dots per nucleus of GFP positive cells was counted (N = 50). Error bars represent standard deviation of the mean from triplicate biological replicates. **p < 0.01 by Student’s t-test.

Considering that the biochemical protocols used to lyse cells and isolate protein complexes from extracts are prone to disrupt *bona fide* protein interactions in cellular nuclei, we next used the *in situ* proximity ligation assay (PLA) as this method is suitable for visualizing interactions between proximally positioned proteins in cells after fixation of large labile complexes. All of the proteins analyzed (BRG1, BAF155, TLX and LSD1) were confirmed to be strictly localized in the nucleus by immunofluorescence assay (Supplementary Fig. S9) and the same antibodies were used for PLA. Similar to the positive control using the anti-BRG1/anti-BAF155 antibody pair (two subunits of SWI/SNF core complex), the anti-BRG1/anti-TLX, anti-BRG1/anti-LSD1, anti-LSD1/anti-TLX, and anti-LSD1/anti-BAF155 antibody pairs detected close localization between these 5 pairs of proteins (Fig. 6). When each single antibody was used, there were only a few signals detected (Fig. 6, Supplementary Fig. S10). When TGS-01 cells transduced with FLAG-tagged DPF1 expression vector were analyzed by PLA, we were able to detect close localization between DPF1 and TLX, DPF1 and LSD1, DPF1 and BRG1, and DPF1 and BAF155 (Supplementary Fig. S11). However, when only anti-FLAG antibody was used, there were only marginal signals. From these results, the corepressor complex including TLX and LSD1 were shown to be closely associated with the SWI/SNF core complex. To examine impacts of *DPF1* knockdown on this larger SWI/SNF complex, TGS-01 cells expressing shDPF1 were analyzed by PLA. The results indicated that signals detecting the proximal localization of BRG1 and TLX or BRG1 and LSD1 were drastically decreased, whereas those for BRG1 and BAF155 or LSD1 and TLX were unaffected (Fig. 7, Supplementary Fig. S12). These observations are consistent with the idea that DPF1 connects the SWI/SNF core complex and corepressor complex containing TLX and LSD1/RCOR2.

## Discussion

We have here described differences in the subunit composition of the SWI/SNF core complex and their associated cofactors between sphere and differentiated monolayer cultures of GICs. We have also shown that among the mRNAs enriched in GIC sphere cultures, DPF1 and DPF3a play key roles in the stemness maintenance of these cells. By shRNA-mediated knockdowns in GICs, both proteins were shown to be essential for growth and sphere formation in culture and tumor propagation in a mouse orthotopic transplantation model (Figs 2 and 4, Supplementary Fig. S3). The effects of *DPF1* or *DPF3a* knockdowns on cellular survival and sphere formation were very rapid, which in itself suggests that DPF1 and DPF3a could be direct therapeutic targets for the suppression of GICs. When TGS-01 cells were transduced with vectors expressing shDPF1 or shDPF3a and transplanted into nude mice, the shDPF1 expressing cells showed much stronger anti-tumor forming activity than those transduced with shDPF3a (Fig. 4). This result is consistent with our findings from the *in vitro* sphere forming assays indicating that a *DPF1* knockdown cannot be significantly rescued by DPF3a cDNA expression, whereas the sphere forming activity of *DPF3a*-knockdown cells can be rescued by DPF1 cDNA expression (Fig. 2). Overall, DPF1 shows potential as a future therapeutic target for GBM.

Coimmunoprecipitation experiments revealed the presence of a large protein complex containing both SWI/SNF core complex and a corepressor complex (Fig. 5, Supplementary Fig. S8). However, a detailed description of this complex was difficult probably because the large complex is fragile. An antibody to the putative component could partly disrupt this complex when it binds to its antigen. It is also possible that the formation and dissociation of this large complex are in equilibrium in a cell. To resolve this problem, we fixed the labile large complexes in cells, and monitored proximally positioned proteins using PLAs (Figs 6 and 7, Supplementary Figs S10, S11 and S12). By this method, we were able to detect a close association between the SWI/SNF core complex and corepressor complex in the nuclei of GICs. The corepressor complex was found to be composed of the nuclear receptor TLX and LSD1/RCOR2 using various different pairs of antibodies. Since TLX is the only transcription factor which can bind directly to DNA in this large complex, we believe that this large complex has a transcriptional suppression function mediated through TLX in a SWI/SNF-dependent manner. In this regard, it is worth noticing that the well-known target genes that are suppressed by TLX, *P21* ^28, 29^ and *BMP4* ^30^, were found to be upregulated upon the induction of GIC differentiation (Supplementary Fig. S13). Considering that the suppression of these two genes have been reported to be essential for stemness maintenance of GICs^31, 32^, they might be the direct targets of the large complex. The model of the large complex formation is schematically represented in Supplementary Fig. S14. From these observations, we believe that TLX plays a crucial function in this large protein complex to maintain GICs.

It is noteworthy that we have previously observed large complex formation between the SWI/SNF core complex and a complex containing NRSF/CoREST/mSin3A in epithelial tumors^33^. A subset of the SWI/SNF core was present in this larger complex and was found to be responsible for the suppression of such neuronal genes as *synaptophysin*, *SCGI* and *synapsin1* in non-neural cells. The representative SWI/SNF core complex is also present and contributes to the basal expression of the *IL6* gene in the same cell.

Shortly after the knockdown of *DPF1*, the stem-cell like properties of the GICs were rapidly suppressed and the proximal locations between BRG1 and TLX or between BRG1 and LSD1 were disrupted, whereas those between BRG1 and BAF155 or between TLX and LSD1 were unaffected (Fig. 7). Although the exact molecular components of the entire complex remain to be resolved, in the large complex detected in our current analyses, DPF1 probably function as linkers between the SWI/SNF core and corepressor.

It should be pointed out in this regard also that a previous search was conducted for candidates for direct regulatory targets (transcription factors and epigenetic regulators) of the four core transcription factors (POU3F2, SOX2, SALL2 and OLIG2) that would mediate stemness maintenance by analyzing core nodes in the transcriptional network controlled by these four core factors^6^. Intriguingly, DPF1 was among those candidates and is suggested to function downstream of OLIG2. Interestingly, some other components in larger SWI/SNF complexes were identified, LSD1 and RCOR2 were also included in the list of candidates. Therefore, we believe that we have here isolated a protein complex that assembles many key regulators to directly epigenetically regulate stemness in GICs. The possible adaptor protein, DPF1, which links the core SWI/SNF and corepressor complexes, is likely to be a very promising therapeutic target for disrupting only the large complex in GICs.

## Materials and Methods

### Cell culture

Three independent glioma initiating cells (GICs) termed TGS-01, TGS-04 and TGS-05 were established as described previously^17^. All human materials and protocols used in this study were approved by the ethics committee of the University of Tokyo Hospital (24-69-250809) and Medical Mycology Research Center (MMRC), Chiba University (#10). All methods were performed in accordance with each university’s guideline and regulation. Informed consent was obtained from all patients. GICs were passaged in DMEM/F12 serum-free medium (Thermo Fisher Scientific) supplemented with B27 (Thermo Fisher Scientific), 20 ng/ml of EGF, and 20 ng/ml of bFGF (both from PeproTech) using ultra-low attachment dishes or flasks. Dulbecco’s modified Eagle’s medium containing 10% fetal bovine serum (FBS) was used to induce the differentiation of GICs and to passage 293FT and PLAT-A human embryonic kidney cells in culture.

### Plasmid construction

For lentivirus construction, the DNA fragment containing *EF1α* promoter region was amplified by PCR from pXL001 (26122^34^, Addgene) using the primer sets listed in Supplementary Table S1 and digested with NheI. pLSP^35^ was digested with ClaI, blunt ended using T4 DNA Polymerase and then digested with XbaI. The resulting 1.5 kb and 5.1 kb fragments were ligated to generate pLE. Pairs of oligonucleotides containing multi cloning sites (MCS) were synthesized as listed in Supplementary Table S1 and inserted into the EcoRV/ClaI sites of pLE to generate pLE-MCS. IRES-EGFP and IRES-Puro^r^ fragments were obtained by PCR from pMXs-IG^36^ and pMXs-IP^36^, respectively, using primer sets listed in Supplementary Table S1 and were digested with XbaI and ClaI. The resulting 1.3 kb and 1.2 kb fragments were inserted into the XbaI/ClaI site of pLE-MCS to generate pLE-IG (EV-2) and pLE-IP (EV-3), respectively.

For shRNA expression vectors, pairs of oligonucleotides encoding gene-specific short hairpin RNA (shRNA) were synthesized as listed in Supplementary Table S1 and inserted into the BbsI/EcoRI sites of pmU6^37^. The pmU6 derivatives shCre#4^38^, shBrm#4^39^, shDPF1-CDS#1^16^ and shDPF3a-3′UTR#2^16^ were previously described. These pmU6-based plasmids were doubly digested with BamHI and EcoRI, and inserted into the same sites of pSSSP^40﻿^, pSSCG^35^ (EV-1) or pLE-IG (EV-2).

For exogenous expression, pairs of oligonucleotides encoding a 3 × FLAG tag were synthesized as listed in Supplementary Table S1, annealed, extended, digested with BglII and MfeI, and inserted into the BglII/MfeI site in MCS of pLE-IG and pLE-IP. DPF1, DPF2, DPF3a, DPF3b, BRG1 and Brm fragments were amplified by PCR using primer sets listed in Supplementary Table S1 and cloned into the BamHI/EcoRI site of pCR2.1. DPF1, DPF2, DPF3a and DPF3b fragments were digested with EcoRI and SalII, and inserted into the MfeI/XhoI site in MCS of pLE-IG and pLE-IP. BRG1 fragment was digested with MfeI and XbaI, and inserted into the MfeI/XbaI site in MCS of pLE-IG and pLE-IP. Brm fragment was digested with EcoRI and XhoI, inserted into the EcoRI/XhoI site in MCS of pLE-IG and pLE-IP. Site-directed mutagenesis was performed using a KOD-Plus-Mutagenesis kit (TOYOBO) in accordance with the manufacturer’s instructions to generate BRG1 shRNA resistant mutant with primer sets listed in Supplementary Table S1. IκBαSR expression vector was kindly gifted by Prof. Shoji Yamaoka^22^. All plasmids were confirmed by DNA sequencing.

### DNA transfection and retro/lentivirus preparation

For the transfection of plasmids into cells, Lipofectamine 2000 (Thermo Fisher Scientific) was used in accordance with the manufacturer’s instructions. Vesicular stomatitis virus-G (VSV-G) pseudotyped retrovirus vectors were produced using the prepackaging cell line PLAT-A. VSV-G pseudotyped lentivirus vectors were produced with the prepackaging cell line 293FT, using the ViraPower Lentiviral Expression System (Thermo Fisher Scientific), in accordance with the manufacturer’s instructions. Three hours after transfection, the medium was changed to virus production serum-free medium (VP-SFM; Thermo Fisher Scientific) containing 4 mM L-glutamine. The transfection supernatant was collected after 24 and 48 hours after transfection, filtered through a 0.45 μm filter, and centrifuged at 6000 × g at 4 °C for 16 hours. The pellets were suspended in culture medium for GICs. For transduction, GICs were incubated with the virus vector stocks at 37 °C for 4 hours.

### RNA preparation and quantitative RT-PCR

Total RNA was extracted using a mirVana microRNA Isolation Kit (Thermo Fisher Scientific). All RNA samples were then treated with TURBO DNase enzyme (TURBO DNA-free Kit; Thermo Fisher Scientific). To detect mRNAs, cDNA was synthesized with a PrimeScript RT Master Mix (TaKaRa Bio) in accordance with the manufacturer’s instructions. Quantitative real-time RT-PCR (qRT-PCR) was performed using a SYBR Select Master Mix (Thermo Fisher Scientific). *GAPDH* mRNA was used as an internal control. The primer pairs used are listed in Supplementary Table S2. qRT-PCRs were performed in triplicate using a StepOne Plus real-time PCR system (Thermo Fisher Scientific).

### Western blotting

Total protein extracts were prepared by boiling the cells in 2 × SDS sample buffer for 10 min at 95 °C. The proteins were then resolved by 10% SDS-PAGE and transferred onto Immobilon-P PVDF membranes (Millipore). Western blotting was performed by incubating the membrane in Can Get Signal Solution I (TOYOBO) containing primary antibodies overnight at 4 °C. After three washes with Tris-buffered saline (TBS) containing 0.1% Tween 20, the membranes were incubated in Can Get Signal Solution II (TOYOBO) containing secondary antibodies [donkey anti-rabbit-horseradish peroxidase (AP182P; Millipore), Peroxidase AffiniPure Donkey Anti-Mouse IgG (715-035-150; Jackson immunoresearch), Peroxidase AffiniPure Donkey Anti-Goat IgG (705-035-147; Jackson immunoresearch) and Anti-DDDDK-tag mAb-HRP-DirecT (M185-7; MBL)] for 1 hour at room temperature (RT). Signals were detected on an AE-9300H-CP Ez-CaptureMG (ATTO) imaging analyzer using ECL Western Blotting Substrate (Promega) or Immunostar DL (WAKO). The primary antibodies used are listed in Supplementary Table S3.

### Single-cell sphere formation assay

Two days after the transduction with shRNA expression retrovirus vectors (pSSCG) or shRNA/cDNA dual expression lentivirus vectors (pLE-IG), GICs were dissociated with TrypLE express (Thermo Fisher Scientific) and GFP (+)/7-AAD (−) cells were sorted by FACS ARIA I or ARIA II (Becton Dickinson) at a density of 1 cell per well into ultra-low attachment 96-well plates (Corning) in 100 μl of DMEM/F12 serum-free medium. After 2 weeks, the percentage of wells containing spheres was calculated.

### Intracranial proliferation assay

Two days after transduction with shRNA expression lentivirus vectors (pLE-IG), GICs were dissociated and GFP (+)/7-AAD (−) cells were sorted by FACS ARIA I, centrifuged and resuspended in DMEM/F12 serum-free medium. A total of 3 × 10^3^ cells (2 μl) were injected stereotactically into the right cerebral hemisphere of 6-week-old female BALB/c nu/nu mice (CLEA Japan) at a depth of 3 mm. All animal experimental protocols were performed in accordance with the policies of the Animal Ethics Committee of the University of Tokyo and performed in compliance with University’s Guidelines for the Care and Use of Laboratory Animals.

### Immunoprecipitation

Cells were lysed with a buffer containing 50 mM Tris-HCl (pH 7.5), 140 mM NaCl, 1 mM MgCl_2_, 0.5 mM DTT, 0.1% Tween20, protease inhibitor cocktail (Nacalai Tesque) and phosphatase inhibitor cocktail (Nacalai Tesque). Immunoprecipitation were performed using Dynabeads Protein G (Thermo Fisher Scientific) in accordance with the manufacturer’s instructions. The antibodies used are listed in Supplementary Table S3.

### Immunofluorescence and proximity ligation assay

For immunofluorescence, GICs were seeded onto an 8-Well Lab-Tek II chamber slide (Nunc) coated with poly-L-lysine and left 5 minutes. The cells were then fixed with PBS containing 4% paraformaldehyde (Nacalai Tesque) for 15 min at RT, washed twice with PBS and permeabilized with 0.5% Triton X-100 PBS for 15 min at RT. After washing twice with PBS, blocking was performed using a 1:1 mixture of 5% BSA, 0.02% NaN3 PBS and Blocking one (Nacalai Tesque) for 1 hour at 37 °C. The samples were then incubated overnight at 4 °C with primary antibodies (listed in Supplementary Table S3) in blocking buffer. The samples were washed twice and subsequently incubated with Alexa Fluor 546 or 488 conjugated secondary antibodies (Thermo Fisher Scientific, 1:1000) in blocking buffer in the dark for 1 hour at RT. The samples were mounted in Vectashield Mounting Medium with DAPI (Vector Laboratories). Fluorescence was detected using a fluorescence microscope (BZ-X710; Keyence). Images were processed using Adobe Photoshop CS3 software.

For the proximity ligation assay (PLA), GICs were seeded, treated and incubated with primary antibodies as described above. The PLA was performed using the Duolink *In Situ* Starter Set ORANGE (Sigma) in accordance with the manufacturer’s instructions. Anti-mouse MINUS and anti-rabbit PLUS PLA probes were used. Fluorescence was detected using a fluorescence microscope (BZ-X710; Keyence).

### Statistical analysis

Results are presented as means ± S.D. Statistical significance for qRT-PCR assays and single-cell sphere formation assay was determined using a two-tailed Student’s t-test. For the survival analysis shown in Fig. 4, differences in survival rates were evaluated by the log-rank test.

## Electronic supplementary material

Supplementary information
