# Author Correction: The role of the SWI/SNF chromatin remodeling complex in maintaining the stemness of glioma initiating cells

**DOI:** 10.1038/s41598-018-31444-z

**Published:** 2018-10-25

**Authors:** Hiroaki Hiramatsu, Kazuyoshi Kobayashi, Kyousuke Kobayashi, Takeshi Haraguchi, Yasushi Ino, Tomoki Todo, Hideo Iba

**Affiliations:** 1 Division of Host-Parasite Interaction, Department of Microbiology and Immunology, The Institute of Medical Science, The University of Tokyo, Tokyo, 108-8639 Japan; 2 Division of RNA Therapy, Medical Mycology Research Center, Chiba University, Chiba, 260-8673 Japan; 3 Division of Innovative Cancer Therapy, and Department of Surgical Neuro-Oncology, The Institute of Medical Science, The University of Tokyo, Tokyo, 108-8639 Japan

Correction to: *Scientific Reports* 10.1038/s41598-017-00982-3, published online 18 April 2017

This Article contains errors in the Reference list. References 10, 15, 16, 27, 33, 35, 38 and 39 were incorrectly given as:

10. Iba, H. D. & Brady, W. F. Obtaining credit reports can be risky business. *J Clin Orthod* **36**, 333–335 (2002).

15. Sakurai, K. *et al*. MicroRNAs miR-199a-5p and -3p target the Brm subunit of SWI/SNF to generate a double-negative feedback loop in a variety of human cancers. *Cancer Res* **71**, 1680–1689, 10.1158/0008-5472.can-10-2345 (2011).

16. Ogata, A. *et al*. Biaryl modification of the 5′-terminus of one strand of a microRNA duplex induces strand specificity. *Bioorg Med Chem Lett* **20**, 7299–7302, 10.1016/j.bmcl.2010.10.077 (2010).

27. Inayoshi, H. & Iba, H. Rhythms Emerge in a Collection of ‘Blind Chemicals’ by the Use of Genetic Switches. *Genome Inform Ser Workshop Genome Inform* **8**, 71–79 (1997).

33. Yamamichi, N. *et al*. Cdx2 and the Brm-type SWI/SNF complex cooperatively regulate villin expression in gastrointestinal cells. *Exp Cell Res* **315**, 1779–1789, 10.1016/j.yexcr.2009.01.006 (2009).

35. Sugimoto, N. & Iba, H. Inference of gene regulatory networks by means of dynamic differential Bayesian networks and nonparametric regression. *Genome Inform* **15**, 121–130 (2004).

38. Ui, M. *et al*. Retrovirus vectors designed for efficient transduction of cytotoxic or cytostatic genes. *Gene Ther* **6**, 1670–1678, 10.1038/sj.gt.3301009 (1999).

39. Yamamichi, N. *et al*. Locked nucleic acid *in situ* hybridization analysis of miR-21 expression during colorectal cancer development. *Clin Cancer Res* **15**, 4009–4016, 10.1158/1078-0432.ccr-08-3257 (2009).

The correct references are listed below as references 1–8.
