# Supplementary material for: The role of the SWI/SNF chromatin remodeling complex in maintaining the stemness of glioma initiating cells
Source: Sci Rep. 2017 Apr 18;7:889. doi: 10.1038/s41598-017-00982-3 (PMC5429847; doi:10.1038/s41598-017-00982-3)
Supplement: Supplementary file 1 — Supplementary information [file 41598_2017_982_MOESM1_ESM.pdf]

Supplementary Information

The role of the SWI/SNF chromatin remodeling complex in maintaining the stemness of glioma initiating cells

Hiroaki Hiramatsu[1,2], Kazuyoshi Kobayashi[1,2], Kyousuke Kobayashi[1], Takeshi Haraguchi[1,2], Yasushi Ino[3], Tomoki Todo[3], and Hideo Iba[1,2,4]

[1]Division of Host-Parasite Interaction, Department of Microbiology and Immunology, The Institute of Medical Science, The University of Tokyo, Tokyo 108-8639, Japan

[2]Division of RNA Therapy, Medical Mycology Research Center, Chiba University, Chiba 260-8673, Japan

[3]Division of Innovative Cancer Therapy, and Department of Surgical Neuro-Oncology, The Institute of Medical Science, The University of Tokyo, Tokyo 108-8639, Japan

[4]To whom correspondence should be addressed. E-mail: iba@ims.u-tokyo.ac.jp.

Supplementary Figure S1: Specific expression of four core transcription factors and other proteins in sphere cultures of GICs and in differentiated monolayer cultures derived from them.

RNA and protein samples were prepared from the parallel cultures of those used for Figure 1a. These samples were then used for qRT-PCR analysis (a) or western blotting analysis (b) of the four core transcription factors and other proteins in GICs maintained in sphere cultures (S) or differentiated monolayer cultures (D). In (a), error bars represent standard deviation of the mean from triplicate experiments. The reduction in mRNA levels of these four genes in each GIC associated with differentiation was statistically significant ($p < 0.01$). In (b), blots in a black line box are originated from the same gel. The same set of protein samples was used for each blot in equal amounts. β-actin was used as the loading control.

Supplementary Figure S2: Suppression of mRNA levels by shRNAs designed for d4-family members, *BRG1* and *Brm*. MDA-MB-231 cells, which express all of the d4-family members, BRG1 and Brm at significant levels were transduced with pSSSP-based retrovirus vectors expressing these shRNAs and control shRNA (shCre#4) as well as an empty vector. mRNA levels were measured by qRT-PCR. The expression levels of cells transduced with the control vector were taken as 1.0 and error bars represent standard deviation of the mean from triplicate experiments.

Supplementary Figure S3: Knockdown effects of d4-family members on the sphere forming activity of TGS-04 and -05 cells.

Relative sphere formation ratio of TGS-04 and TGS-05 cultures transduced with lentivirus based on pLE-IG expressing shRNAs or empty vector (EV-2; lane 1); shDPF1-3'UTR#4 (lane 2), shDPF2-3'UTR#3, #4 and #6 (lanes 3-5), shDPF3a-3'UTR#4 (lane 6) and shDPF3b-CDS#6 and #7 (lanes 7, 8). Error bars represent the standard deviation of the mean from triplicate experiments. **$p < 0.01$ by Student's t-test.

Supplementary Figure S4: Proviral structures of the dual lentiviral vectors, pLE-IG and pLE-IP, simultaneously expressing shRNA and the corresponding mRNA.

Proviral structures of the dual lentiviral vectors; pLE-IG (a, b, c) and pLE-IP (d). mRNA expression is driven by the *EF1α* pol II promoter, whereas that of shRNA is driven by *mU6* pol III promoter. Each mRNA was designed to be resistant to the corresponding shRNA. pLE-IG and pLE-IP vectors that lack the cDNA insertion (a) or shRNA expression unit (b, d) were also used to express only mRNA or shRNA, respectively. Empty vectors (EV-2; pLE-IG, EV-3; pLE-IP) have neither the cDNA nor shRNA expression unit. ΔU3, the U3 sequence from which major enhancer sequences were deleted; R, lentiviral R sequence; U5, lentiviral U5 sequence; Ψ, lentiviral packaging signal.

Supplementary Figure S5: Effects of exogenous expression of d4-family proteins, BRG1 and Brm on the sphere forming activity of TGS-01 cells.

(a) Percentage of sphere forming TGS-01 cells transduced with lentivirus based on pLE-IG expressing d4-family proteins, BRG1 or Brm, or with an empty vector (EV-2). Error bars represent the standard deviation of the mean from triplicate experiments. No statistically significant differences were observed compared with the control (EV-2) ($p > 0.05$). (b) From parallel cultures used in (a) total proteins were prepared and analyzed by western blotting using anti-FLAG antibody. β-actin was used as the loading control. The position of protein markers (#161-0374; BIO-RAD) were indicated.

Supplementary Figure S6: Relationship between NF-κB activity and stem cell properties of GICs.

(a) Percentage of sphere forming TGS-01 cells transduced with lentivirus based on pLE-IG expressing IκBαSR or with an empty vector (EV-2). Error bars represent standard deviation of the mean from quadruplicate experiments. NS = not significant. (b). Immunofluorescent assays were performed to examine subcellular localization of RelA and RelB in sphere cultures of TGS-01 cells. Nuclei were counterstained with DAPI. Scale bar indicates 10 μm.

Supplementary Figure S7: Full-length images of the western blotting corresponding to Figure 5.

Figure 5a, 5b, 5c and 5d were derived from full-length gel images shown in (a), (b), (c) and (d), respectively. Blots in a black line box are originated from the same gel. In gels in a blue broken line box, the same set of protein samples was charged. Arrowheads indicate the position of the bands corresponding to each protein and arrows indicate the position of protein markers (#161-0374; BIO-RAD).

Supplementary Figure S8: Detection of SWI/SNF complex/TLX interactions in GICs.

TGS-04 and TGS-05 lysates were immunoprecipitated with TLX antibodies and the resulting immunoprecipitates were analyzed by western blotting.

Supplementary Figure S9: Subcellular localization of BRG1, BAF155, TLX and LSD1.

Immunofluorescent assays were performed to detect BRG1, TLX, LSD1 and BAF155 in TGS-01 cells. The antibodies used were described for the PLA experiments in Figure 6. Nuclei were counterstained with DAPI. Scale bar indicates 10 μm. Rb; rabbit, Ms; mouse.

Supplementary Figure S10: PLA analysis using single antibodies.

TGS-01 cells were fixed and incubated with a single antibody as follows: anti-BAF155, anti-LSD1 (Rb; rabbit), and anti-LSD1 (Ms; mouse). Nuclei were counterstained with DAPI (blue). The red fluorescence images were obtained using quick-full-focus function of the BZ-X710 (Keyence) at depth of about 10 μm. Scale bar indicates 10 μm.

Supplementary Figure S11: Proximal localization of 3×FLAG-DPF1 with TLX, LSD1, BAF155 or BRG1 in TGS-01 cells exogenously expressing 3×FLAG-DPF1 as detected by PLA.

TGS-01 cells exogenously expressing 3×FLAG-DPF1 were fixed and incubated with a single or pair of antibodies. Red dots indicate interactions and nuclei were counterstained

with DAPI (blue). The red fluorescence images were obtained using quick-full-focus function of the BZ-X710 (Keyence) at depth of about 10 μm. Scale bar indicates 10 μm.

Supplementary Figure S12: Knockdown effects of d4-family members on the proximal localization between SWI/SNF core complex and TLX/LSD1 in TGS-01 cells as detected by PLA.

TGS-01 cells transduced with lentivirus vectors based on pLE-IG expressing shDPF1-3'UTR#4, shDPF2-3'UTR#3 or shDPF3a-3'UTR#4 or an empty vector (EV-2) were fixed three days after the transduction and incubated with pairs of antibody indicated. The number of dots per nucleus of GFP positive cells was counted. Error bars represent 95% confidence intervals of the mean (N = 50).

Supplementary Figure S13: Expression patterns of TLX target genes in sphere cultures of GICs and in differentiated monolayer cultures derived from them.

The expression levels of *BMP4* (a), and *p21* (b) in both sphere and differentiated monolayer cultures of GICs were analyzed by qRT-PCR and compared. Error bars represent the standard deviation of the mean from triplicate experiments. The increase in mRNA levels of these genes in each GIC associated with differentiation was statistically significant ($p < 0.01$).

Supplementary Figure S14: Schematic representation of the model of formation of a larger SWI/SNF complex that is required for stemness maintenance of GICs.

The SWI/SNF core complex and a corepressor complex containing TLX, RCOR2, LSD1 and HDAC2 are linked through d4-family proteins and form a large complex.

Supplementary Table S1. List of primer pairs used for plasmid constructions.

shDPF1-3'UTR#4-sense	5’-TTTGAATTAACTTGTTCTGTGTATGCTTCCTGTCACATACACAGAACAAGTTAATTCTTTTTG-3’
shDPF1-3'UTR#4-antisense	5’-AATTCAAAAAGAATTAACTTGTTCTGTGTATGTGACAGGAAGCATACACAGAACAAGTTAATT-3’
shDPF2-3'UTR#3-sense	5’-TTTGTAGCTTCACCTTGTTATTCCGCTTCCTGTCACGGAATAACAAGGTGAAGCTACTTTTTG-3’
shDPF2-3'UTR#3-antisense	5’-AATTCAAAAAGTAGCTTCACCTTGTTATTCCGTGACAGGAAGCGGAATAACAAGGTGAAGCTA-3’
shDPF2-3'UTR#4-sense	5’-TTTGCTCTTAACTGAATTGGGAGCGCTTCCTGTCACGCTCCCAATTCAGTTAAGAGCTTTTTG-3’
shDPF2-3'UTR#4-antisense	5’-AATTCAAAAAGCTCTTAACTGAATTGGGAGCGTGACAGGAAGCGCTCCCAATTCAGTTAAGAG-3’
shDPF2-3'UTR#6-sense	5’-TTTGGTGATCACAGGGTTCAAACAGCTTCCTGTCACTGTTTGAACCCTGTGATCACCTTTTTG-3’
shDPF2-3'UTR#6-antisense	5’-AATTCAAAAAGGTGATCACAGGGTTCAAACAGTGACAGGAAGCTGTTTGAACCCTGTGATCAC-3’
shDPF3a-3'UTR#4-sense	5’-TTTGAAATCGAAGCAATATCCTGTGCTTCCTGTCACACAGGATATTGCTTCGATTTCTTTTTG-3’
shDPF3a-3'UTR#4-antisense	5’-AATTCAAAAAGAAATCGAAGCAATATCCTGTGTGACAGGAAGCACAGGATATTGCTTCGATTT-3’
shDPF3b-CDS#6-sense	5’-TTTGGGAACTGCTCAAAGAGAAAGGCTTCCTGTCACCTTTCTCTTTGAGCAGTTCCCTTTTTG-3’
shDPF3b-CDS#6-antisense	5’-AATTCAAAAAGGGAACTGCTCAAAGAGAAAGGTGACAGGAAGCCTTTCTCTTTGAGCAGTTCC-3’
shDPF3b-CDS#7-sense	5’-TTTGATGACCAGCTACTCTTCTGCGCTTCCTGTCACGCAGAAGAGTAGCTGGTCATCTTTTTG-3’
shDPF3b-CDS#7-antisense	5’-AATTCAAAAAGATGACCAGCTACTCTTCTGCGTGACAGGAAGCGCAGAAGAGTAGCTGGTCAT-3’
shBrm#8-sense	5’-TTTGTGATAAACTACAAAGATAGGGCTTCCTGTCACCCTATCTTTGTAGTTTATCACTTTTTG-3’
shBrm#8-antisense	5’-AATTCAAAAAGTGATAAACTACAAAGATAGGGTGACAGGAAGCCCTATCTTTGTAGTTTATCA-3’
shBRG1-CDS#2-sense	5’-TTTGTTGGAAGTACATGATTGTGGGCTTCCTGTCACCCACAATCATGTACTTCCAACTTTTTG-3’
shBRG1-CDS#2-antisense	5’-AATTCAAAAAGTTGGAAGTACATGATTGTGGGTGACAGGAAGCCCACAATCATGTACTTCCAA-3’
shBRG1-CDS#4-sense	5’-TTTGCGTATCGCGGCTTTAAATACGCTTCCTGTCACGTATTTAAAGCCGCGATACGCTTTTTG-3’
shBRG1-CDS#4-antisense	5’-AATTCAAAAAGCGTATCGCGGCTTTAAATACGTGACAGGAAGCGTATTTAAAGCCGCGATACG-3’

EF1α-Fwd	5'-GTTTAAACGCCACAAATGGCAGTATTCATCCA-3'
EF1α-Rev	5'-AAAGCTAGCATCGATGATATCCTCACGACACCTGAAATGGAAGA-3'
MCS-sense	5'-ATCAGATCTCAATTGCTCGAGGCGGCCGCCAGCTGTCTAGACAT-3'
MCS-antisense	5'-CGATGTCTAGACAGCTGGCGGCCGCCTCGAGCAATTGAGATCTGAT-3'
IRES-EGFP-Fwd	5'-AAATCTAGAGGCCGCTACGTAAATTCCG-3'
IRES-EGFP-Rev	5'-AAAATCGATGCTCGACTTACTTGTACAGCTCGTCCATG -3'
IRES-Puro^r-Fwd	5'-AAATCTAGACGGCCGCTACGTAAATTCCG-3'
IRES-Puro^r-Rev	5'-AAAATCGATGCTCGATCAGGCACCGGGCTTGCGGGT-3'
3×FLAG-sense	5'-AAAAAGATCTACTACCATGGACTACAAAGACCATGACGGTGATTATAA AGATCATGACAT-3'
3×FLAG-antisense	5'-TTTTCAATTGCTTGTCATCGTCATCCTTGTAGTCGATGTCATGATCTTTAT AATCACCGT-3'
DPF1-Fwd	5'-GAATTCATGGGCGGCCTCAGCGCCCGCCCGA-3'
DPF1-Rev	5'-GTCGACGGTATCGATAAGCTTCTAGGTGAGGGTGATGTAAGC-3'
DPF2-Fwd	5'-GAATTCATGGCGGCTGTGGTGGAGAAT-3'
DPF2-Rev	5'-GTCGACGGTATCGATAAGCTTTCAAGAGGAGTTCTGGTTCTGG-3'
DPF3a/b-Fwd	5'-GAATTCATGGCGACTGTCATTCACAAC-3'
DPF3a-Rev	5'-GTCGACGGTATCGATAAGCTTTTAGCAACTGCCCTTTTTATCTG-3'
DPF3b-Rev	5'-GTCGACGGTATCGATAAGCTTCTAGGCCTGGCAGCCAAA-3'
BRG1-Fwd	5'-CAATTGATGTCCACTCCAGACCCA-3'
BRG1-Rev	5'-TCTAGAGTCAGTCTTCTTCGCTGCCA-3'
Brm-Fwd	5'-GAATTCATGTCCACGCCCACAGACCC-3'
Brm-Rev	5'-CTCGAGTCACTCATCATCCGTCCCAC-3'
BRG1(mutation)-Fwd	5'-ATATGATCGTCGACGAAGGTCACCGCATGAAGAAC-3'
BRG1(mutation)-Rev	5'-ATTTCCATCTTATCTTGGCGAGGATGTGCTTGTCT-3'

Supplementary Table S2. List of primer pairs used for qRT-PCR.

Gene	Forward primer	Reverse primer
GAPDH	CTCTGCTCCTCCTGTTCGAC	TTAAAAGCAGCCCTGGTGAC
Brm	CAGAAGCAGAGCCGCATCA	GGCCTGAAGTCTGTATTCCCG
BRG1	AGATGTCTTCCGGGCCA	AGCTGGTTCTGGTTAAATGGG
INI1	GACGCCTTCACCTGGAACA	CGTCAGCGGGTTCAAATCCA
BAF155	TGACAGAGCAGACCAATCACA	AGAACTCAGGAAGAGCACGC
BAF170	ACGGCAAGAACAAGTCCAAGA	GGCAGGCGGTAGAGGTAAGA
BAF60A	TGGTAGAATGGCACAGGACCG	GGTAATCCAGCATCAGTAGGACA
BAF60B	AGGCGTACATGGATCTCTTGG	GCTGGGACTGAACGTATTGGA
BAF60C	TCATCAGCGTGGACCCTTCA	TTGGCCGTGGATAGGAGGAA
BAF57	GGTCACGGCATCCTCTGGTA	TCTCCCACAACTTTAGGTCAGG
ARID1A	CAGATGGGACACCCAAGACA	GTCCAGAGGTTTCCTACCCAC
ARID1B	CGACTCTACGTCTGCGTCAA	CGTTTAGGTTGGTTGCCAGC
BAF53A	ACAGTGGAACGGAGGTTTAGC	GGGAACTCTTTCTCAAGGGCA
BAF53B	CGTCAAGTCTGAGCCAAACC	GCAGGAATGTTGTACTGCTCG
DPF1	CCGGAAGGGAGCTGGA	CAGGTAGGCGAGCACCAC
DPF2	CAGAGGAACAGGGAAGATGGC	ACTCCGGTCTGTGAGTCCAA
DPF3a	TCAGACAACACAGGAGCCAG	AACTGAGGCCATTCCCAAGG
DPF3b	AGCTACTCTTCTGCGATGACTG	TTCTCTTTGAGCAGTTCCCAGC
SS18	GATGAACGGCCAGATGCCTG	TGATGATGGCACAGAATGGTTG
CREST	AACATGCAGTCCAACCCAGTCTC	CCTGCGCCGAGCTGTAGTG
BRD9	CTGCTCTACTCAGCCTACGG	GCATCCTTCACAAACTCCTGC
BCL7A	AGGCAAGGACGAGAAGTGTG	GCTGGAGTTGCTGCTGTTC
BCL7B	AAGTGGGTGACTGTGGGTGA	AGGCATCAGAAGGAAAGCCA
BCL7C	GGCCAAGAGAGAGATCCCG	CCTCAGCTTCCAGCAGTTC
BCL11A	CGCCGCAAGCAAGGCAAA	CGTGGTCTGGTTCATCATCTGTAA
BCL11B	ATGGGGAGAGAAGGAGACTGAA	CGGCTGACGGTTACTTAGGAC
POU3F2	GCCCTCTTGTTCCCTCTCTAA	ACACATCATTACACCTGCTACC
SOX2	GAGGGGTGCAAAAGAGGAGA	CGTGAGTGTGGATGGGATTG
SALL2	TCTTCCACCTTTACCACCCAC	AGATGAGGCGAGGCAATCAG
OLIG2	ACACAAATGGTAAACTCCTCCA	ACACGGCAGACGCTACAAA
BMP4	GGATCTTTACCGGCTTCAGTC	GGGATGTTCTCCAGATGTTCTTC
p21	GCAGACCAGCATGACAGATTTC	ATGTAGAGCGGGCCTTTGAG

Supplementary Table S3. List of primary antibodies used in this study.

Application	Name	Host	Supplier	Catalog No.	Dilution
Western blotting	anti-DPF1	Goat	Abcam	ab3940	1/500
	anti-DPF2	Rabbit	Abcam	ab128149	1/2000
	anti-DPF3	Rabbit	original[19]	-	1/1000
	anti-SOX2	Rabbit	Abcam	ab92494	1/500
	anti-SALL2	Rabbit	Bethyl	A303-208A	1/2500
	anti-POU3F2	Goat	Santa Cruz	sc-6029	1/200
	anti-OLIG2	Rabbit	Millipore	AB9610	1/500
	anti-β-actin	Mouse	Santa Cruz	sc-47778	1/5000
	anti-BRG1	Rabbit	Santa Cruz	sc-10768	1/200
	anti-Brm	Rabbit	Abcam	ab15597	1/1000
	anti-BAF155	Goat	Santa Cruz	sc-9747	1/500
	anti-LSD1	Rabbit	Abcam	ab129195	1/1000
	anti-RCOR2	Rabbit	Santa Cruz	sc-102078	1/200
	anti-TLX	Mouse	Perseus Proteomics	PP-H6506-00	1/1000
	anti-HDAC2	Rabbit	Santa Cruz	sc-7899	1/200
Immunoprecipitation	normal rabbit IgG	Rabbit	Santa Cruz	sc-2027	2μg/sample
	normal mouse IgG	Mouse	Santa Cruz	sc-2025	2μg/sample
	anti-TLX	Mouse	Perseus Proteomics	PP-H6506-00	2μg/sample
	anti-LSD1	Rabbit	Abcam	ab17721	2μg/sample
	anti-RCOR2	Rabbit	Abcam	ab37113	2μg/sample
Immunofluorescence and proximity ligation assay	anti-RelA	Rabbit	Cell Signaling	#8242	1/100
	anti-RelB	Rabbit	Abcam	ab33907	1/100
	anti-BRG1	Mouse	Santa Cruz	sc-17796	1/50
	anti-BAF155	Rabbit	Abcam	ab72503	1/200
	anti-TLX	Rabbit	Santa Cruz	sc-292096	1/50
	anti-LSD1	Rabbit	Abcam	ab17721	1/200
	anti-LSD1	Mouse	Cell Signaling	#4218	1/100
	anti-FLAG	Mouse	Sigma-Aldrich	F1804	1/1000
	anti-FLAG	Rabbit	Cell Signaling	#14793	1/200

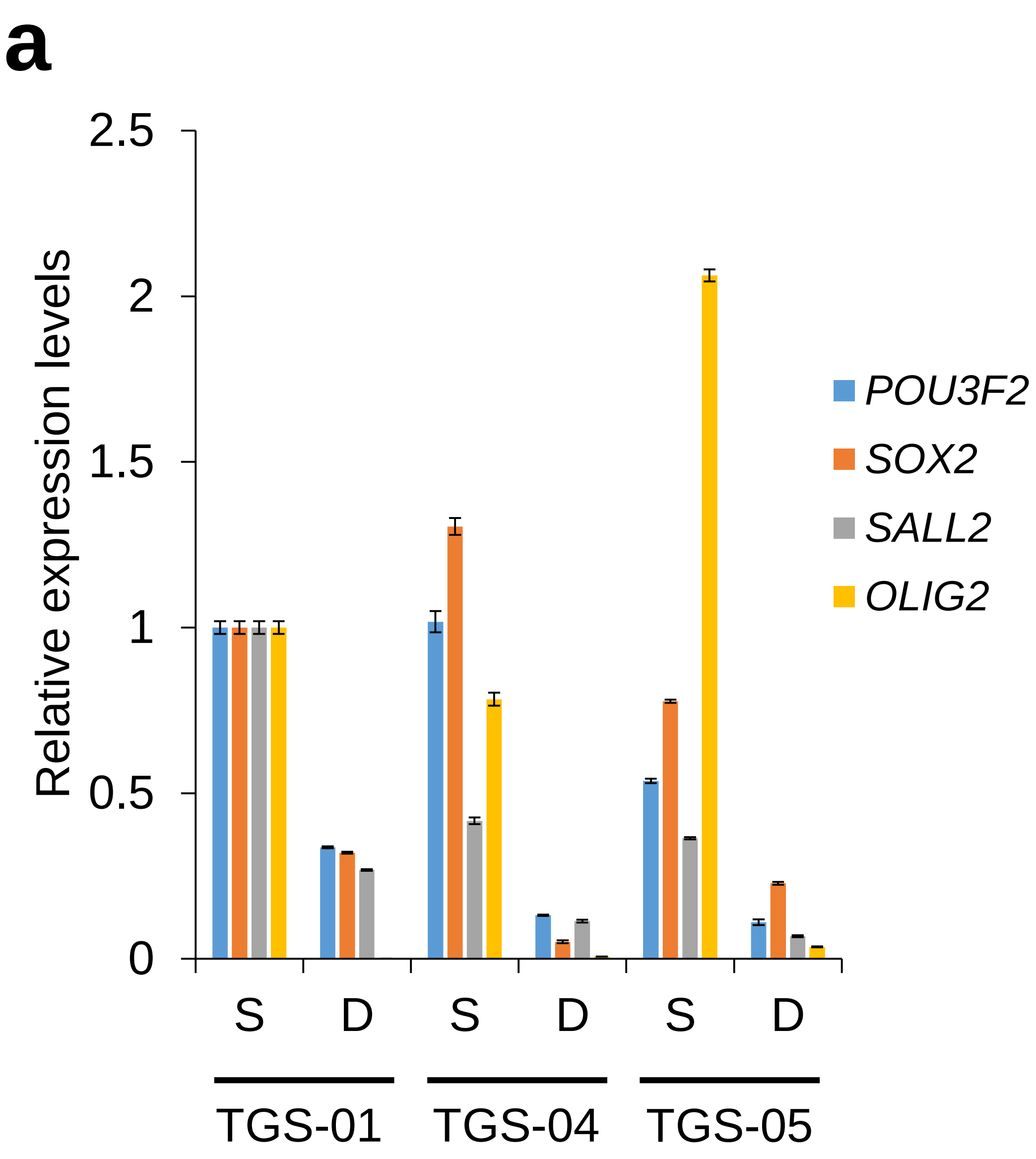

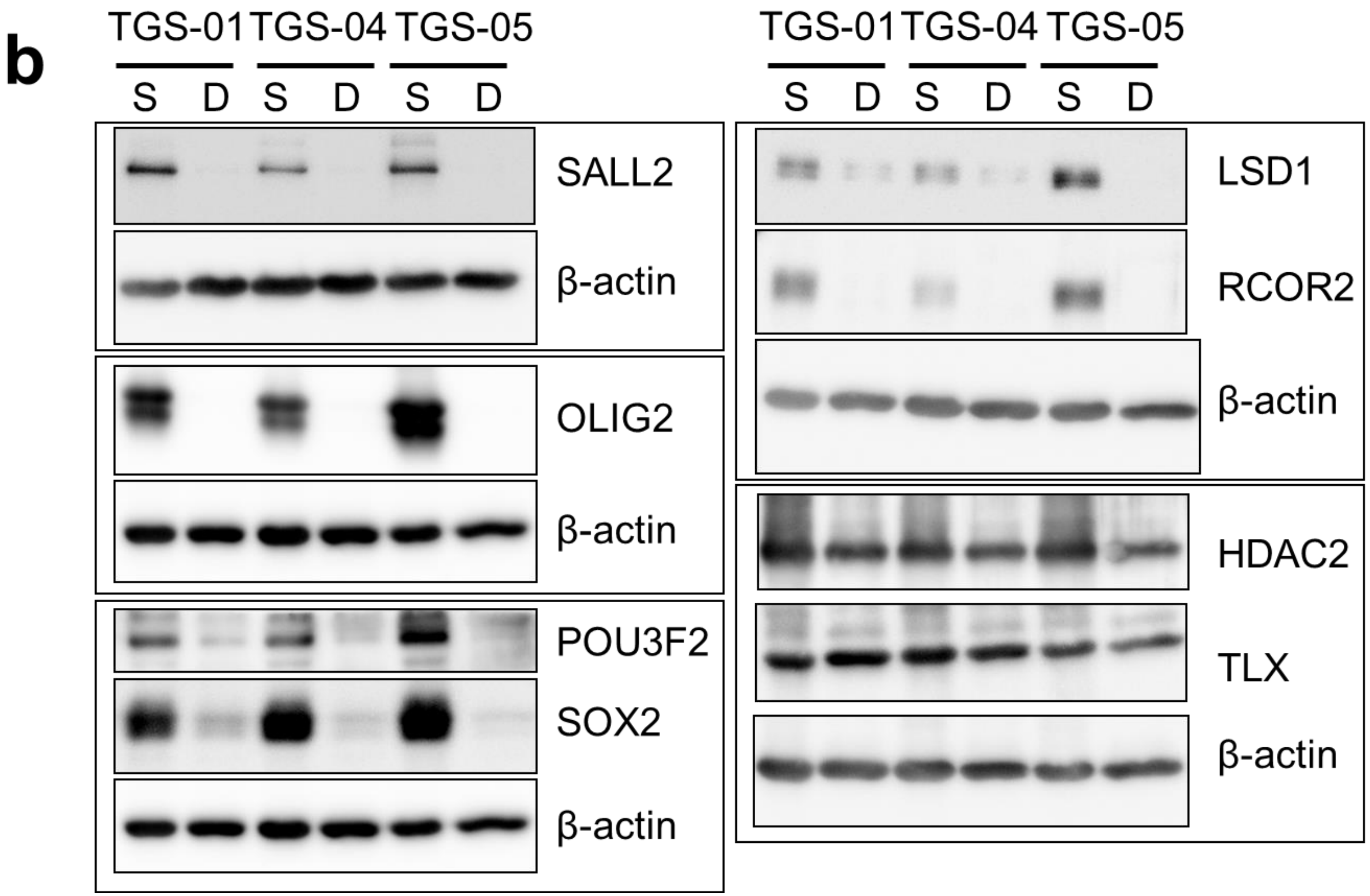

Supplementary Figure 1

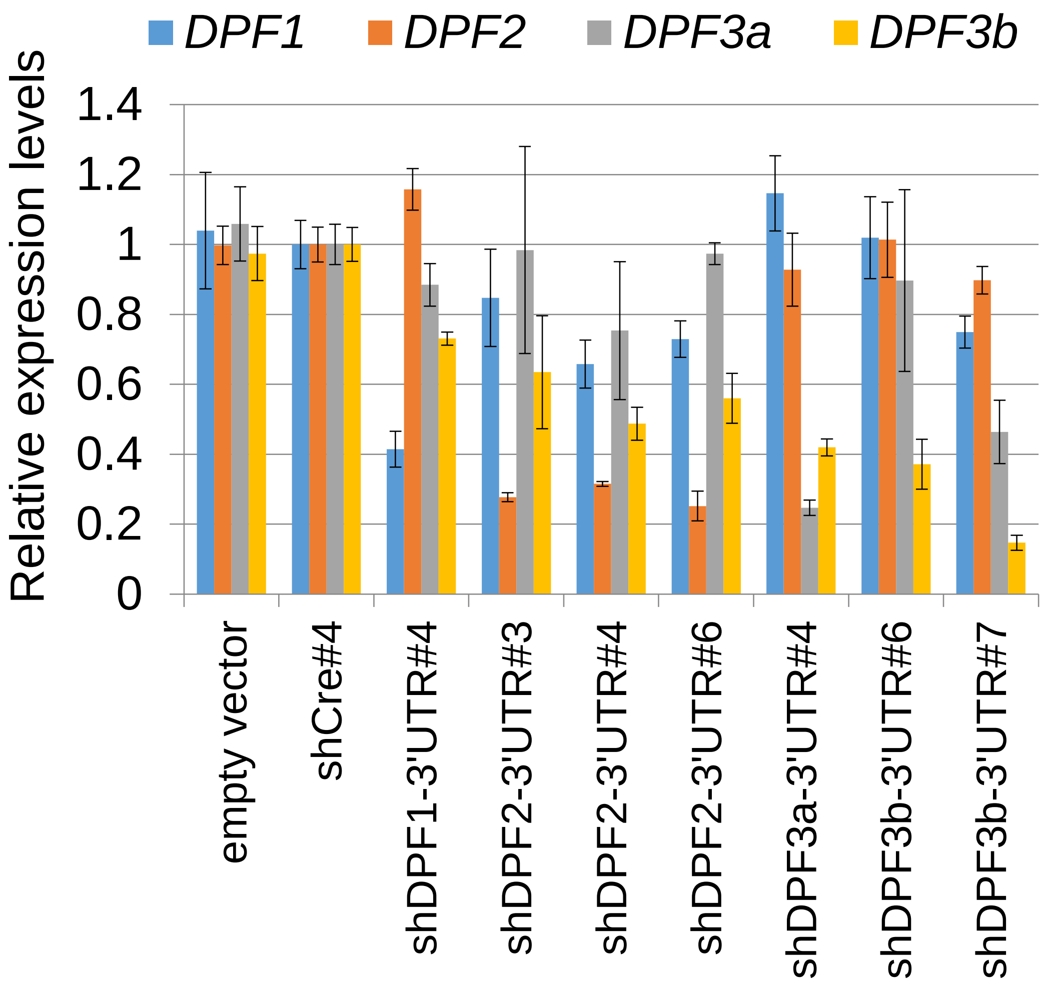

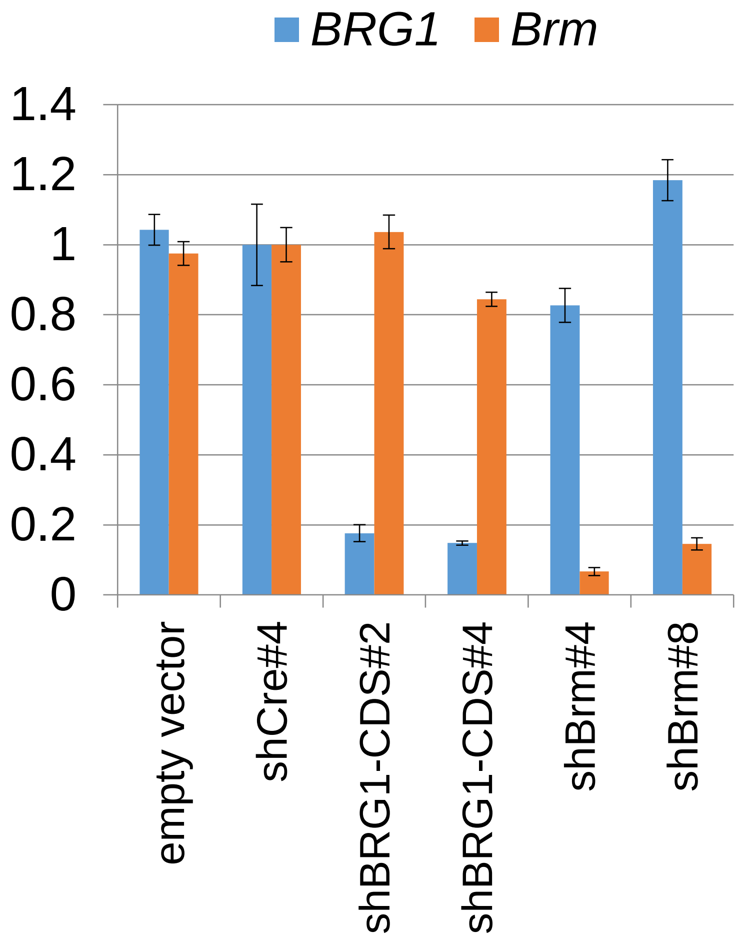

Supplementary Figure 2

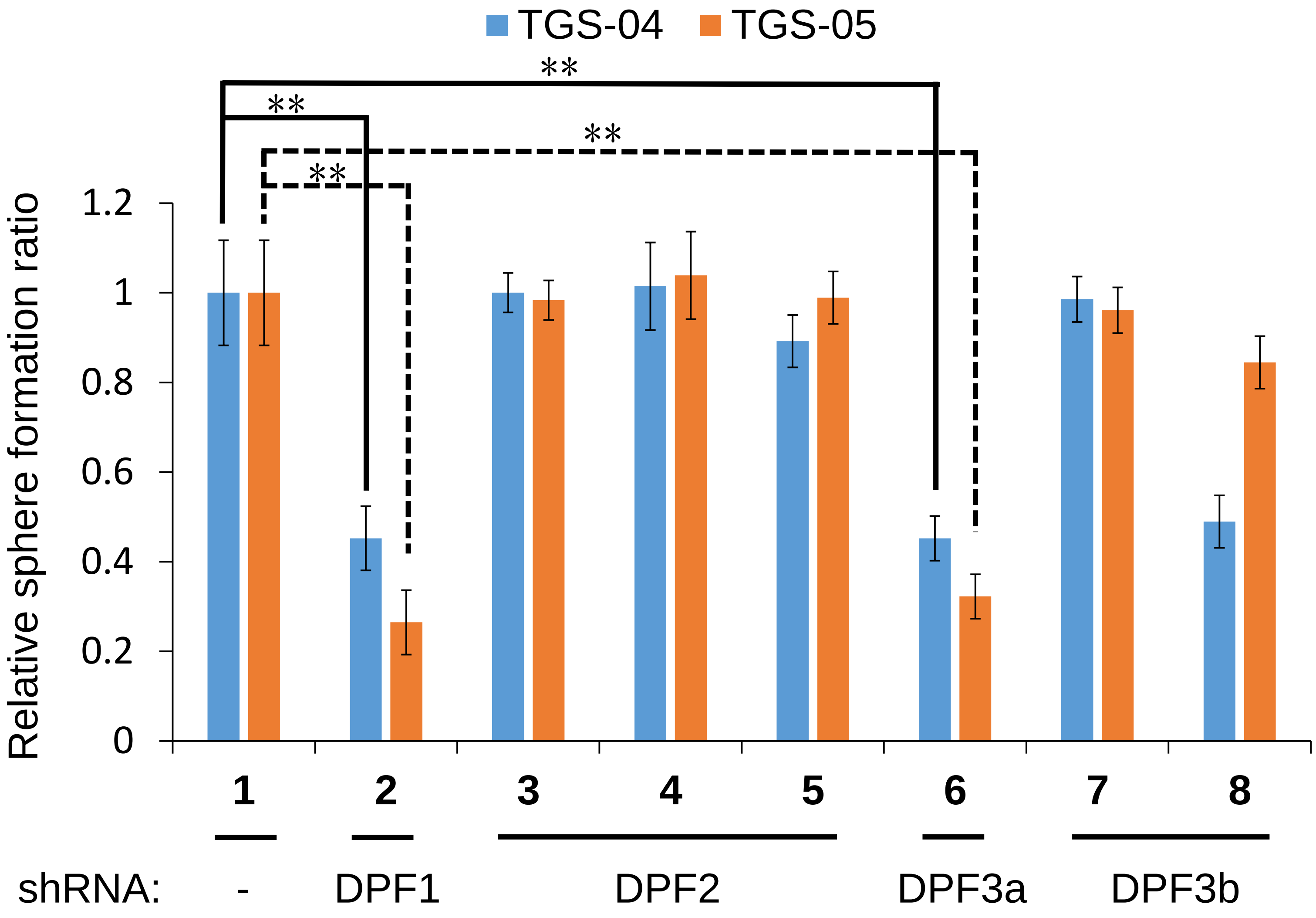

Supplementary Figure 3

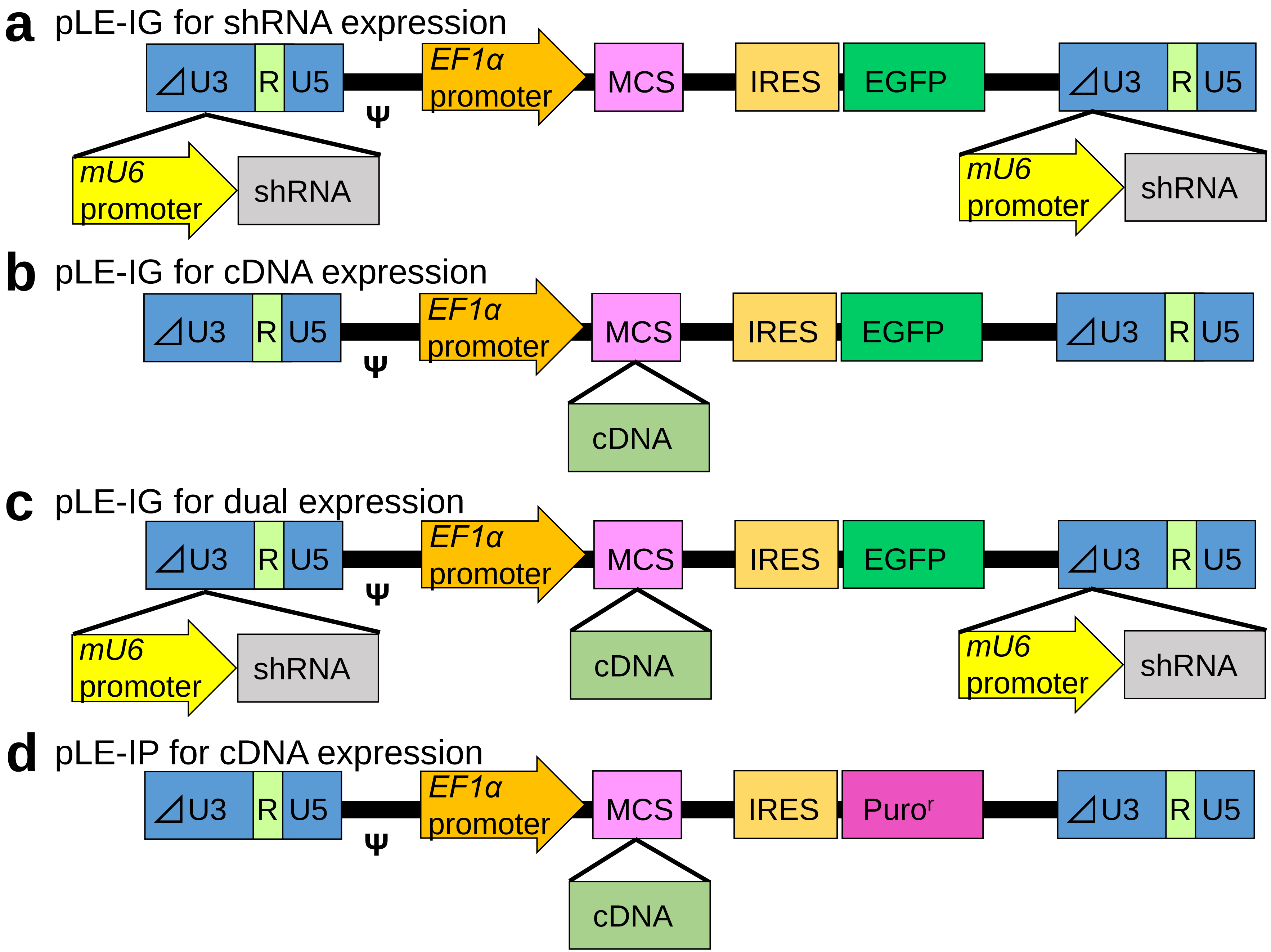

Supplementary Figure 4

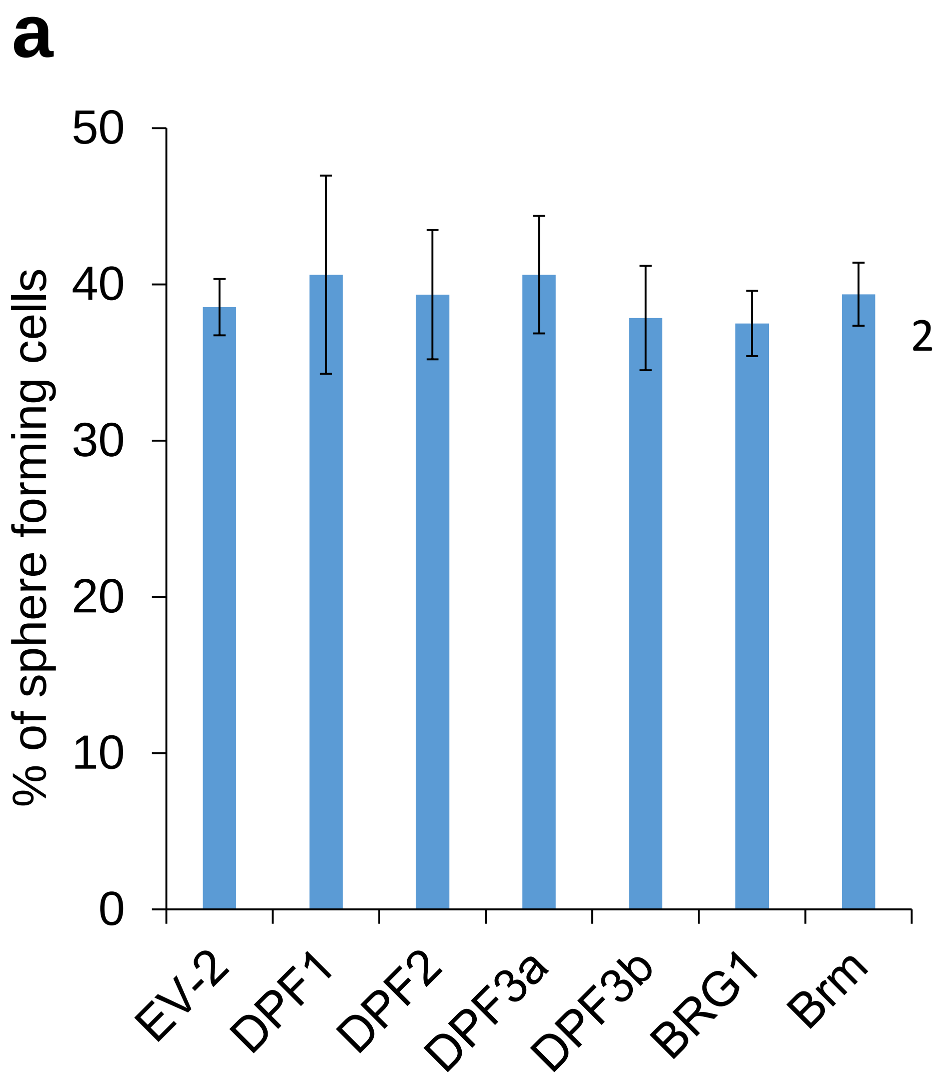

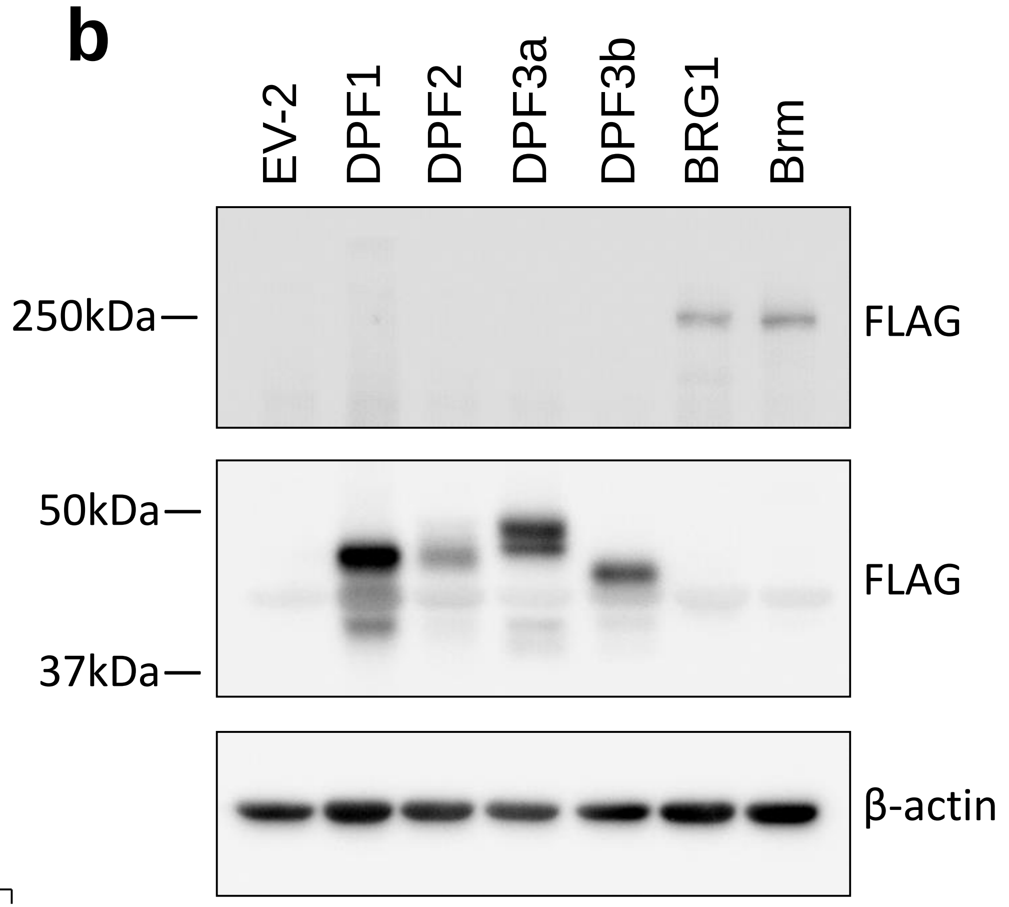

Supplementary Figure 5

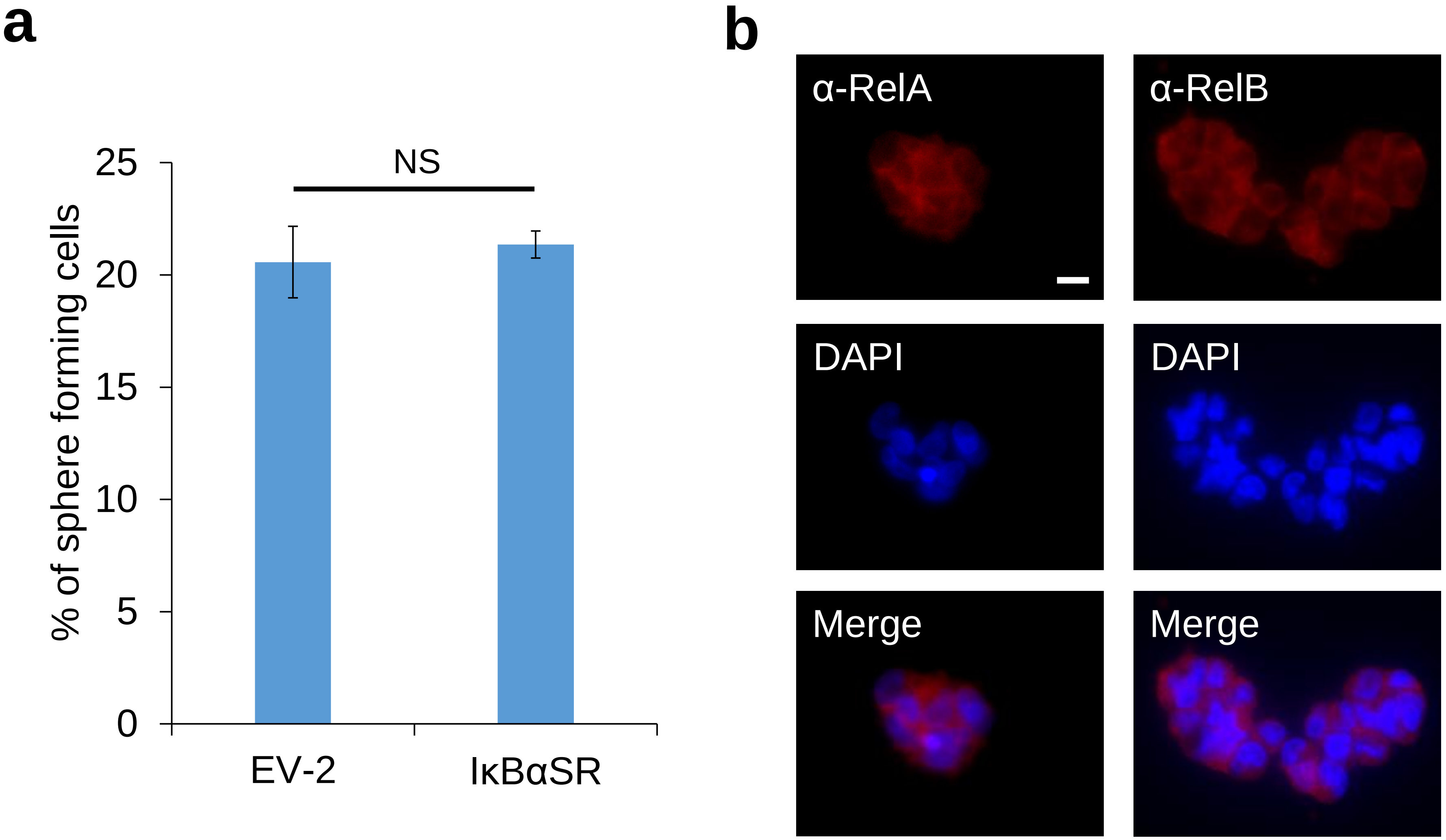

Supplementary Figure 6

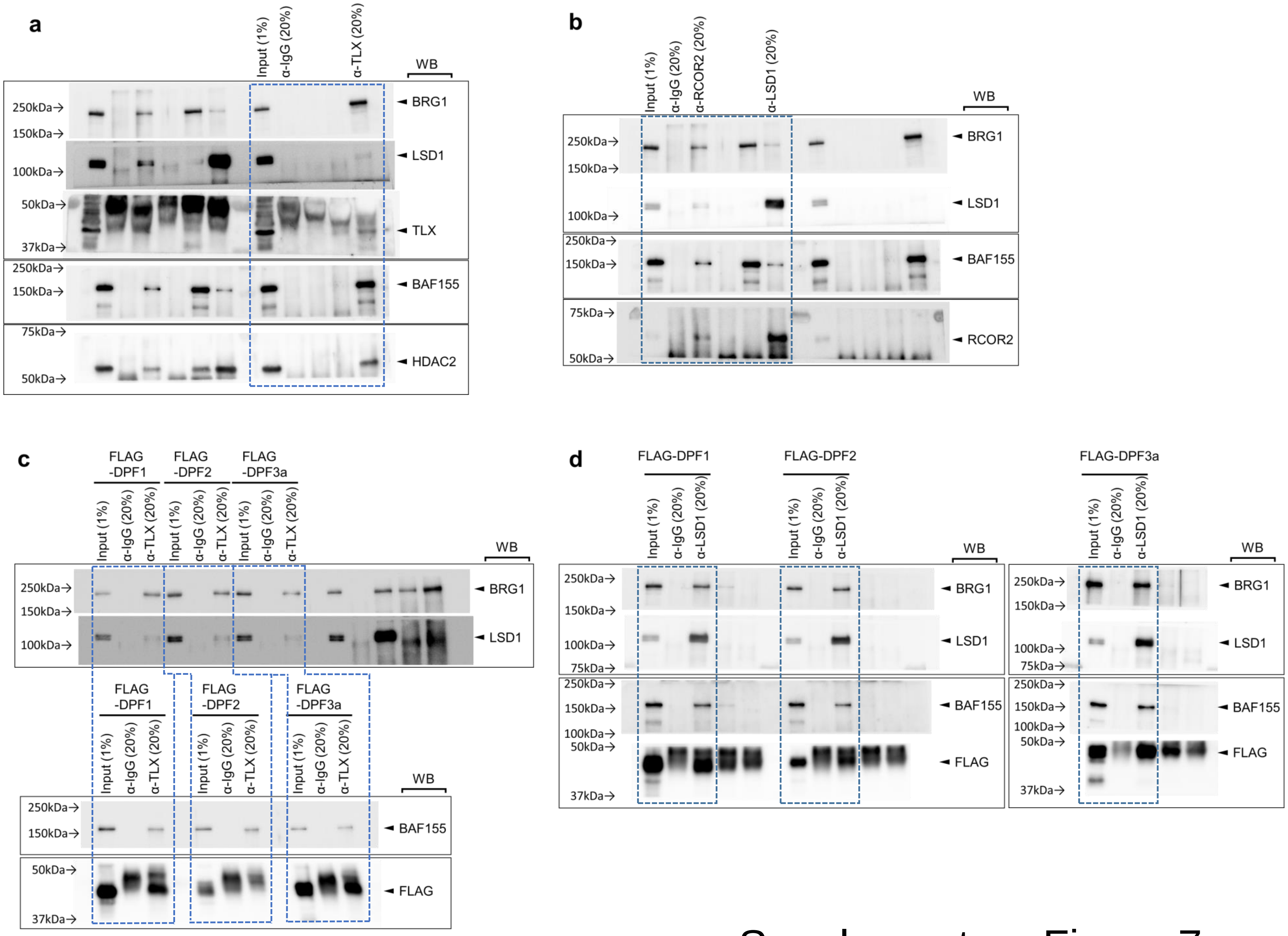

Supplementary Figure 7

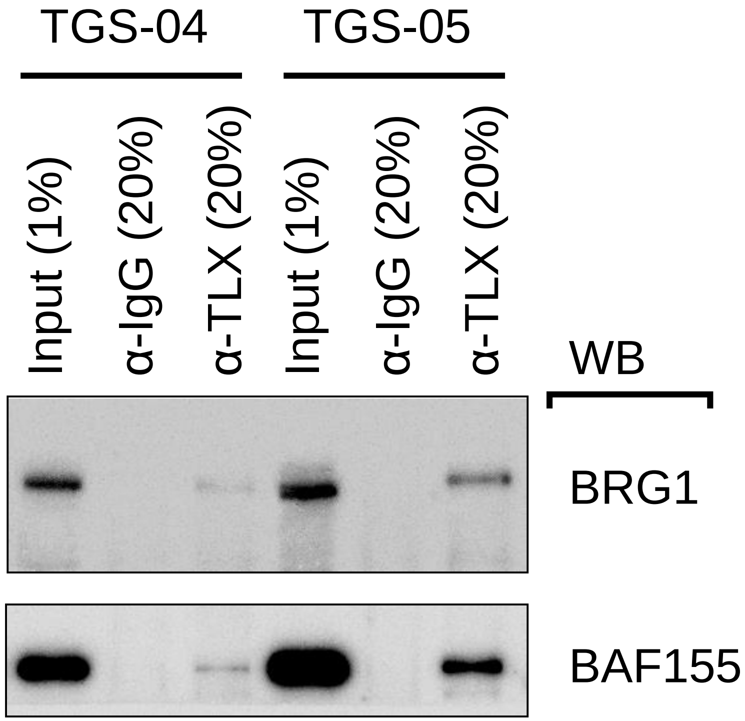

Supplementary Figure 8

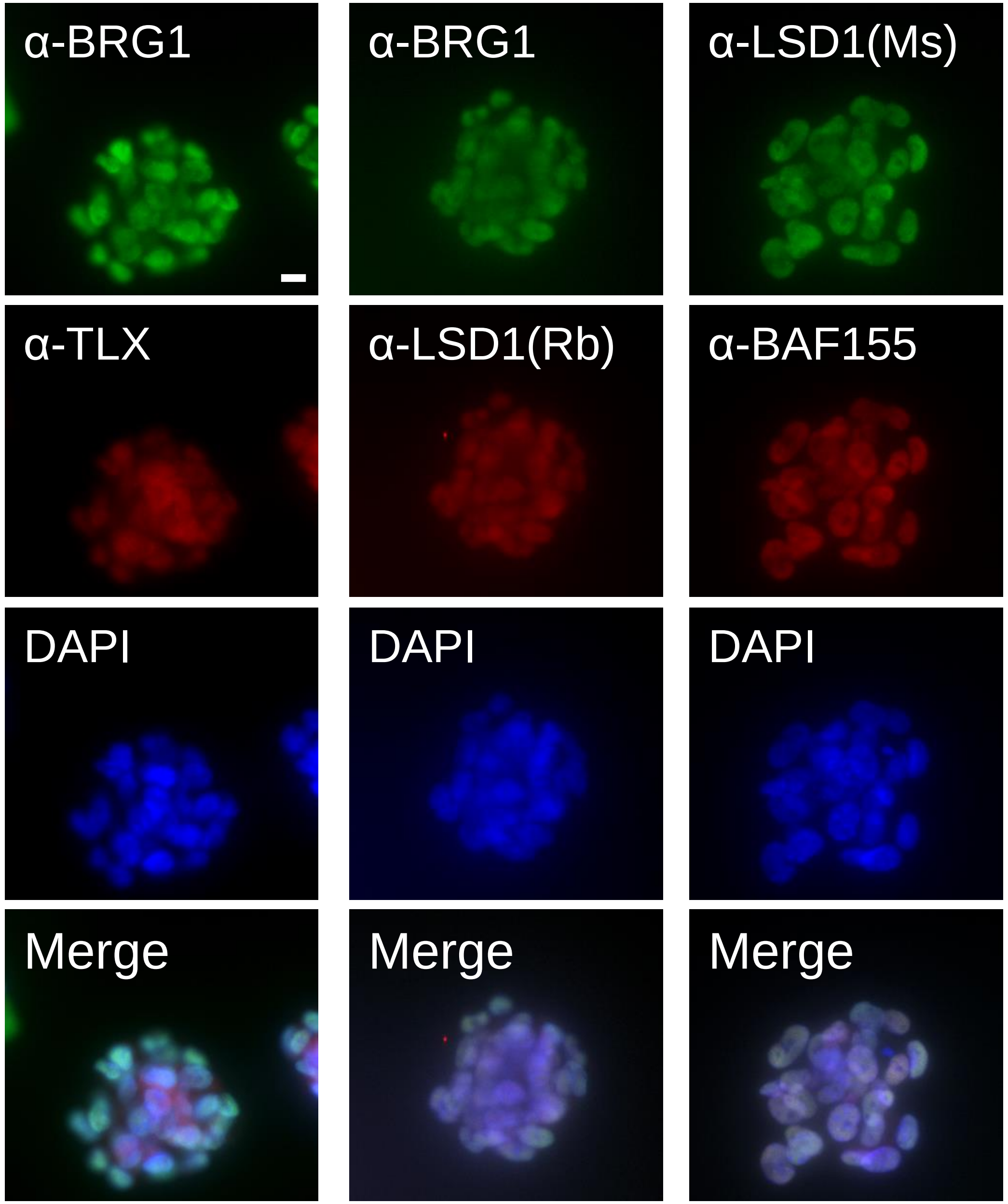

Supplementary Figure 9

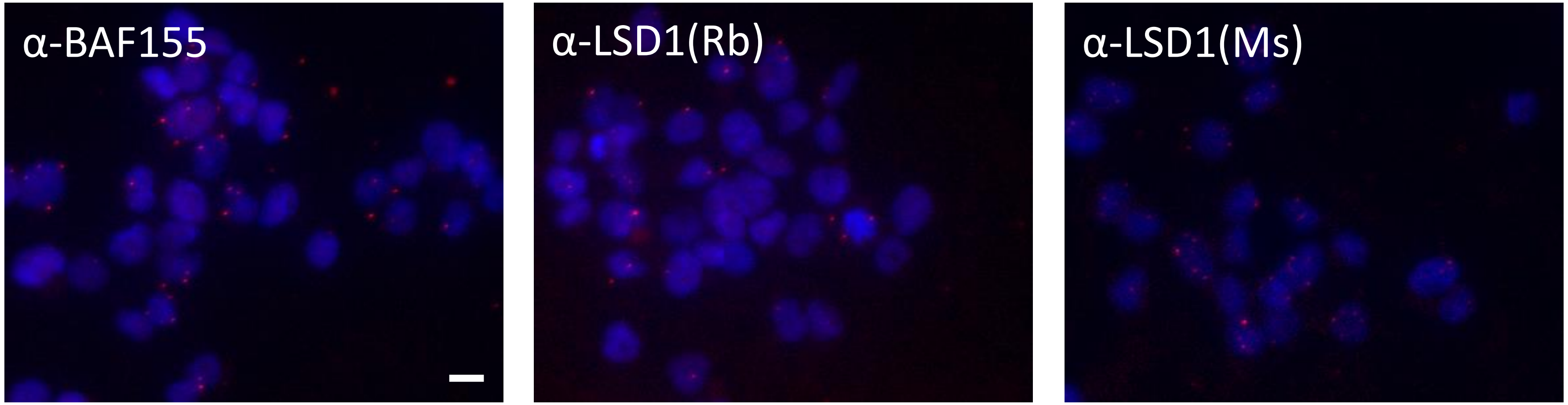

Supplementary Figure 10

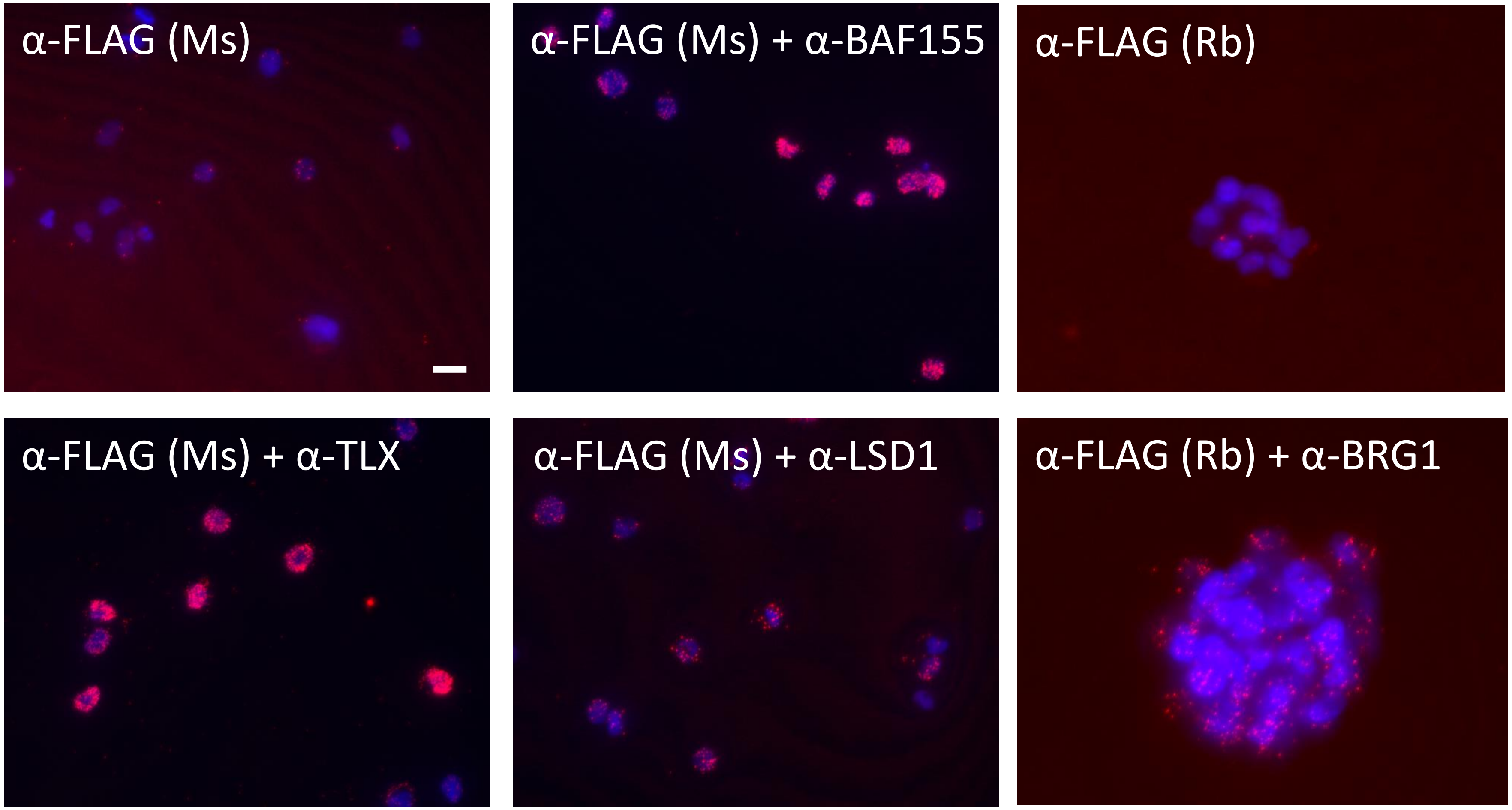

Supplementary Figure 11

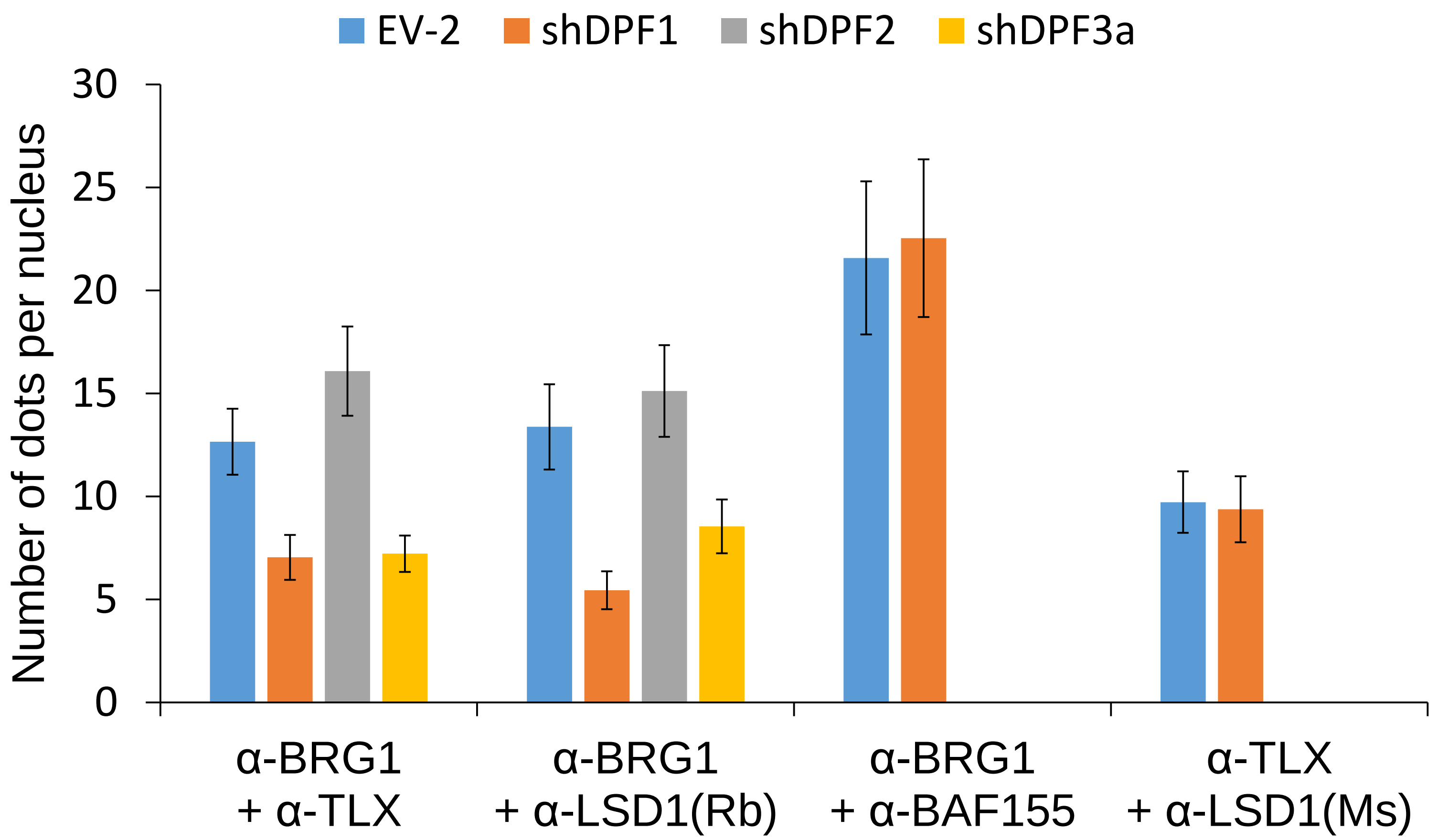

Supplementary Figure 12

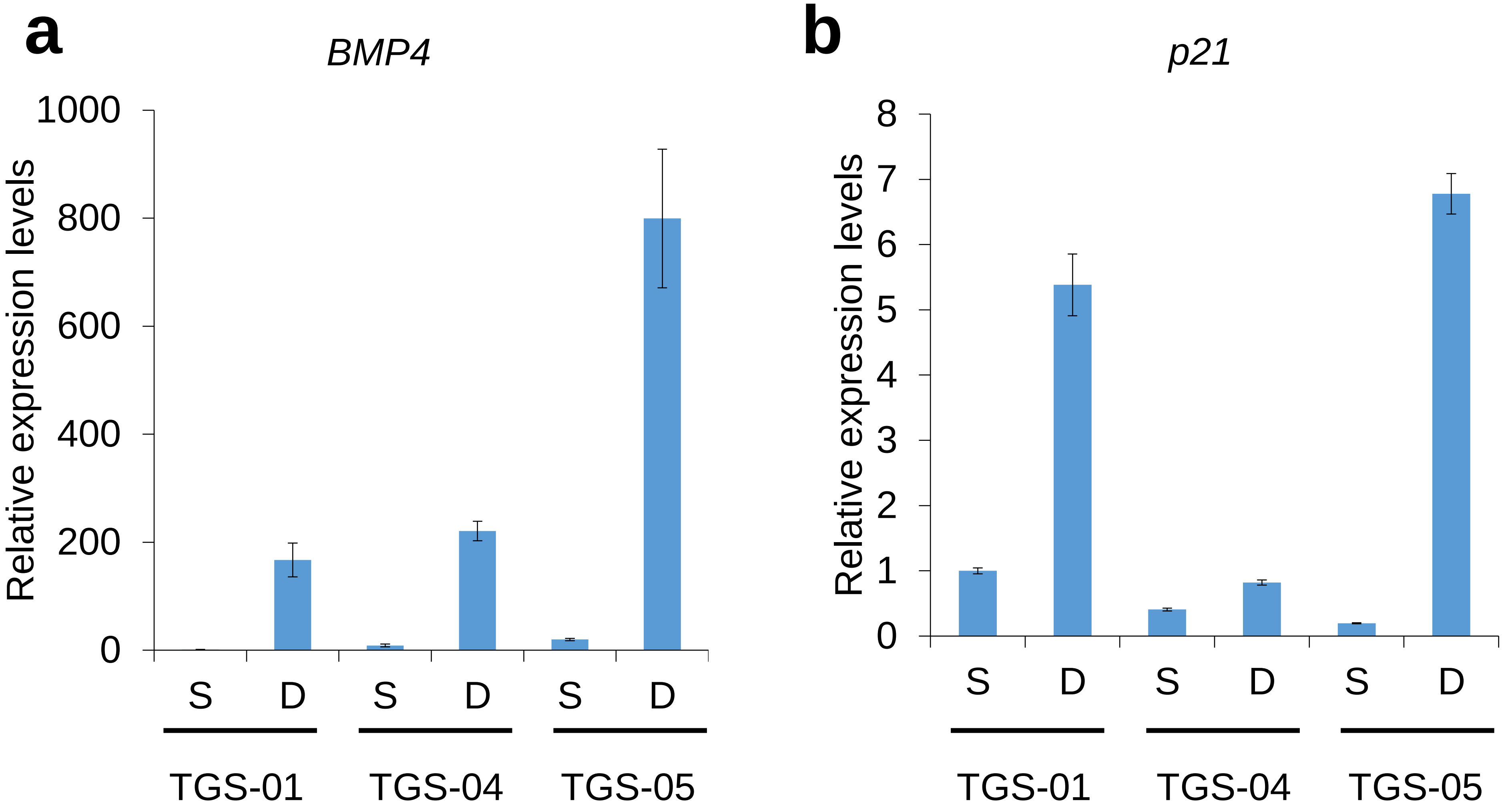

Supplementary Figure 13

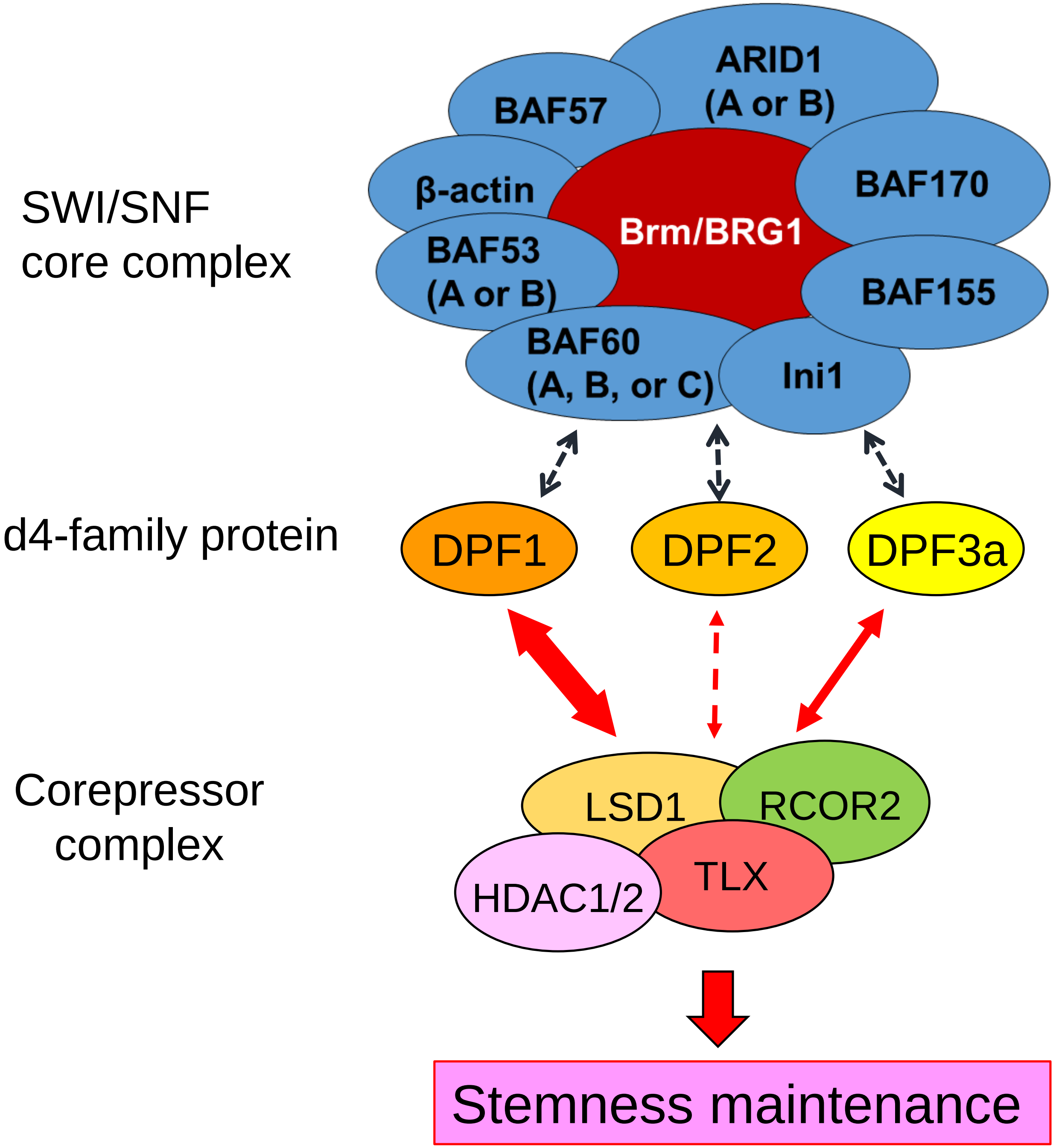

Supplementary Figure 14
